# The relationship between pathogen life‐history traits and metapopulation dynamics

**DOI:** 10.1111/nph.17948

**Published:** 2022-01-22

**Authors:** Laura J. A. van Dijk, Johan Ehrlén, Ayco J. M. Tack

**Affiliations:** ^1^ Department of Ecology, Environment and Plant Sciences Stockholm University SE‐106 91 Stockholm Sweden

**Keywords:** alternate hosts, life‐history traits, *Ochropsora ariae*, Plant–pathogen interactions, spatiotemporal disease dynamics, *Synchytrium anemones*, *Tranzschelia anemones*, *Urocystis anemones*

## Abstract

- Plant pathogen traits, such as transmission mode and overwintering strategy, may have important effects on dispersal and persistence, and drive disease dynamics. Still, we lack insights into how life‐history traits influence spatiotemporal disease dynamics.
- We adopted a multifaceted approach, combining experimental assays, theory and field surveys, to investigate whether information about two pathogen life‐history traits – infectivity and overwintering strategy – can predict pathogen metapopulation dynamics in natural systems. For this, we focused on four fungal pathogens (two rust fungi, one chytrid fungus and one smut fungus) on the forest herb *Anemone nemorosa*.
- Pathogens infecting new plants mostly via spores (the chytrid and smut fungi) had higher patch occupancies and colonization rates than pathogens causing mainly systemic infections and overwintering in the rhizomes (the two rust fungi). Although the rust fungi more often occupied well‐connected plant patches, the chytrid and smut fungi were equally or more common in isolated patches. Host patch size was positively related to patch occupancy and colonization rates for all pathogens.
- Predicting disease dynamics is crucial for understanding the ecological and evolutionary dynamics of host–pathogen interactions, and to prevent disease outbreaks. Our study shows that combining experiments, theory and field observations is a useful way to predict disease dynamics.

## Introduction

Pathogens are well‐known for their complex life cycles and diversity of life‐history traits (Seifert & DiRita, 2006). These life cycles and traits are expected to play a major role in the ecology and evolution of plant–pathogen interactions (Barrett *et al*., 2008; Burdon & Thrall, 2014). For example, transmission mode (van Doremalen *et al*., 2020), infectiousness (Chowell *et al*., 2006), virulence (Anderson & May, 1982) and overwintering strategy (Soper, 1929) all have been predicted to strongly affect disease dynamics and host–pathogen coevolution (Burdon & Laine, 2019). Empirically assessing the relationship between pathogen life‐history traits and their disease dynamics is a crucial next step to enhancing the predictability of disease distributions, and, ultimately, to gain a better understanding of the ecology and evolution of host–pathogen interactions in natural and agricultural systems (Thrall & Burdon, 1997). However, few studies have focused on the relationship between life‐history traits and disease dynamics, and most studies on spatial disease dynamics have targeted single pathogen species (Laine & Hanski, 2006; Soubeyrand *et al*., 2009; Smith *et al*., 2011). As a result, we currently rely mainly on modelling studies and cross‐system comparisons for inferences about the relationship between pathogen life‐history traits and disease dynamics (Thrall & Burdon, 1999; El Jarroudi *et al*., 2020). One way to explore the relationship between pathogen life histories and disease dynamics is to first use experiments to quantify life‐history traits of several pathogens sharing the same host, and then use theory to formulate hypotheses on how differences in traits are expected to influence disease dynamics that can be tested in a field‐based setting.

Plants are host to a wealth of fungal pathogens with distinct life histories. For example, whereas some fungal pathogens cause only local infections, others are systemic, meaning that their mycelium can spread internally from the site of infection to other plant organs (Burdon, 1987; Wennström, 1999). In some cases, this allows a pathogen to overwinter within the twigs or roots of its host plant, causing recurrent infection in the next growing season (Wennström & Ericson, 1992, 1994). Moreover, systemic infection might allow for horizontal transmission through clonal growth of the plant and subsequent propagation of the pathogen (Wennström, 1994), and vertical transmission through seeds (Abdelfattah *et al*., 2021), whereas dispersal of spores through air or water allows for longer dispersal distances (Brown & Hovmøller, 2002). Pathogens also vary in their ability to infect different life stages of the host plant (Burdon, 1987; Kranz, 1990), and may infect seedlings (Bell *et al*., 2006; Broders *et al*., 2007), adult plants (Wennström & Ericson, 1990) or both (Arseniuk *et al*., 1993). Lastly, although some pathogens spend their entire life cycle on one host species, others need multiple hosts in order to complete their life cycle (Duplessis *et al*., 2021). For example, the stem rust pathogen *Puccinia graminis*, a major threat to wheat production, needs barberry as an alternative host to complete its life cycle consisting of five spore stages (de Bary, 1865). Because life‐history traits can determine how, when and where pathogens infect their host, they are likely to influence disease dynamics, and it is important to investigate relationships between these traits and disease dynamics for a diverse set of pathogens. Such insights also then may be used to generate predictions on the spread and invasiveness of pathogens within natural and agricultural systems.

In order to predict how traits of pathogens can influence spatial and temporal disease dynamics when hosts are patchily distributed, one useful framework is metapopulation theory (Antonovics, 2004; Jousimo *et al*., 2014). Some aspects of metapopulation dynamics are expected to be influenced only relatively little by the specific set of traits that a species possess. For example, larger patches are predicted to be occupied more often by pathogens than smaller patches, because colonization events and rescue effects are more likely to occur, and extinction events will be less frequent (Hanski, 1994; Brooks *et al*., 2008). Other aspects of metapopulation dynamics might be more dependent on pathogen traits. For example, pathogens with long‐distance spore dispersal may be more likely to colonize isolated host patches, and thus their distribution is less limited by spatial connectivity of host patches than pathogens with more limited dispersal abilities (Thrall & Burdon, 1999). For pathogens utilizing multiple host species, distribution across host patches is expected to depend on the spatial distribution of all hosts (Jokela & Lively, 1995; Perea *et al*., 2020). Besides dispersal across patches, the transmission mode of the pathogen also may affect spread of disease within a patch. For example, spore dispersal may lead to higher within‐patch disease intensities than systemic spread of disease via clonal reproduction, such as rhizomes (Piqueras, 1999; Thrall & Burdon, 1999).

In this study, we combined experimental assays, metapopulation theory and field surveys to investigate to what extent life‐history traits of pathogens can explain the spatial and temporal dynamics of diseases. As a model system, we used the four major fungal pathogens, *Ochropsora ariae*, *Tranzschelia anemones*, *Synchytrium anemones* and *Urocystis anemones* on a perennial herb, the wood anemone *Anemone nemorosa*. In order to link the traits of these pathogens to metapopulation dynamics in the field, we used a three‐step approach. First, we conducted an experiment to investigate pathogen life‐history traits. Specifically, we were interested in the infectivity of pathogen spores for seedlings and adult plants, and the ability of pathogens to reside in the plant’s rhizome during winter and cause recurrent infection. Second, based on the identified traits of each pathogen, we constructed pathogen‐specific hypotheses on metapopulation dynamics. Third, we tested these hypotheses by following the spatial and temporal distributions of the four diseases in 81 to 139 plant patches over four years in the field.

## Materials and Methods

### Study system

The wood anemone *Anemone nemorosa* (Ranunculales, Ranunculaceae) is a perennial herb that is widely distributed across European forests, with its range limit in northern Scandinavia (Shirreffs, 1985). Wood anemones grow in patchily distributed populations across forest floors. They spread clonally by growth of their rhizomes, and reproduce sexually via seeds (Ernst, 1983). Seed fertility differs strongly among anemone populations and habitats (3–82%) (Canullo, 1985), and anemones do not build up a seed bank (Shirreffs, 1985; Holderegger, 1996). Compared to other clonal species, *A*. *nemorosa* has a relatively high genetic variation and clonal diversity within and among populations, especially at distances of > 0.5 m (Stehlik & Holderegger, 2000). Anemones have a stem height of *c*. 10 cm, and typically have three leaves with lobes (Shirreffs, 1985; van Dijk *et al*., 2021). In Sweden, anemones usually flower from the end of April to the beginning of June. At the end of the growing season, aboveground vegetative parts die back, and only rhizomes persist to the next season. Anemone rhizomes are *c*. 5–15 cm in length on average (García‐Guzmán & Wennström, 2001), and vegetative spread through growth of rhizomes is slow (*c*. 2 cm radial spread per year) (Shirreffs & Bell, 1984). Anemone seeds have no mechanism for long‐distance dispersal (Holderegger, 1996; Brunet & von Oheimb, 1998). The mean migration rate of anemones in Sweden via seed dispersal has been estimated at 20 cm per year (Brunet & von Oheimb, 1998).

The wood anemone is infected by a diversity of pathogens, and its four major fungal pathogens in Sweden are *Ochropsora ariae*, *Tranzschelia anemones*, *Synchytrium anemones* and *Urocystis anemones* (henceforth referred to by their genus names), which are all specialized on anemone in Sweden (Hylander *et al*., 1953; Lindeberg, 1959). Ochropsora (Pucciniales, Uropyxidaceae) is a rust fungus with a putative perennial systemic lifestyle, which can spread via the rhizomes of anemones. Anemones infected with Ochropsora typically have elongated stems, and rarely produce a flower (García‐Guzmán & Wennström, 2001; van Dijk *et al*., 2021). Ochropsora infects two hosts to complete its sexual life cycle. In spring, Ochropsora infects anemones, producing white aeciospores on the underside of anemone leaves. The fungus then infects the rowan, *Sorbus aucuparia*, on which asexual urediniospores and then teliospores form, which mature during autumn before the leaves drop. Ochropsora generally infects rowan seedlings and saplings, and a previous study observed rowan to always be ≤ 90 cm from anemones infected with Ochropsora (García‐Guzmán & Wennström, 2001). Basidiospores infect the rhizomes of anemone from the leaf litter during the next spring (Gjærum, 1974; García‐Guzmán & Wennström, 2001). Like Ochropsora, Tranzschelia (Pucciniales, Uropyxidaceae) is a rust fungus with a putative perennial systemic lifestyle, which elongates the shoot and inhibits flowering of the infected host (García‐Guzmán & Wennström, 2001; van Dijk *et al*., 2021). Tranzschelia is microcyclic and has two spore stages (teliospores and basidiospores) on a single host (*A*. *nemorosa*), and can survive as mycelium in the rhizome buds of anemones (Scholler *et al*., 2019). Tranzschelia causes brown lesions with teliospores on the underside of anemone leaves. Synchytrium (Chytridiomycota, Chytridiales) is a chytrid fungus with a putative nonsystemic lifestyle, causing purple‐brown lesions on the leaves and stems of anemones. In spring, motile zoospores disperse via water film surfaces to infect anemones. At the end of the growing season, sexual resting spores are formed, which potentially disperse long distances via air (Hampson, 1996). Urocystis (Urocystidales, Urocystidaceae) is a smut fungus with a putative annual systemic lifestyle. Infected anemones show blisters on the leaves and stems that burst open towards the end of the growing season to release teliospores. Teliospores drop to the soil and overwinter, and in spring basidiospores germinate to infect anemones.

### Life‐history traits of pathogens

In order to investigate two traits assumed to be important for metapopulation dynamics (i.e. the ability of spores to infect seedlings and plants, as well as ability to overwinter in plant rhizomes) of four fungal pathogens on the wood anemone, we conducted pathogen inoculation experiments with seeds and rhizomes of anemones in a common garden. Towards the end of the growing season, we collected seeds and rhizomes from anemones without visible disease symptoms (henceforth referred to as ‘healthy anemones’), as well as rhizomes from anemones diseased with Ochropsora, Tranzschelia, Synchytrium and Urocystis (based on presence of visible disease symptoms). We collected all plant material from wild populations at the same location (forests around Stockholm University) in June 2019. We filled trays (20 × 30 cm) with potting soil (Krukväxtjord; SW Horto, Hammenhög, Sweden), in which we sowed seeds in approximately regular densities (*c*. 1000 seeds per tray), or planted six to eight rhizomes with the same infection status, with each rhizome measuring 6–8 cm in length and with one growing bud. Some of these trays then were inoculated with spores, by burying parts of infected leaves collected from the field within the top 2 cm of the soil. For Tranzschelia, Synchytrium and Urocystis spores, we added infected leaves of the wood anemone, collected at the end of May. For Ochropsora spores, we added infected leaves of the alternate host, *S*. *aucuparia*, collected at the end of September. We took care to inoculate trays directly after collection of leaf material, or otherwise store plant material for a maximum of one night at 4°C to prevent desiccation of leaves and spores. Inoculations resulted in six different treatments: (1) ‘seeds’ (five trays, sowed seeds); (2) ‘seeds + spores’ (five to eight trays per pathogen, sowed seeds were inoculated with spores); (3) ‘rhizomes’ (10 trays, healthy rhizomes); (4) ‘rhizomes + spores’ (10 trays per pathogen, healthy rhizomes were inoculated with spores); (5) ‘infected rhizomes’ (10 trays per pathogen, rhizomes from previously infected plants); (6) ‘infected rhizomes + spores’ (10 trays per pathogen, rhizomes from previously infected plants were inoculated with spores of the same pathogen), resulting in a total of 165 trays (Supporting Information Fig. S1). These treatments were used to investigate: (1) The infectivity of overwintering spores from collected leaves, both for germinating seedlings (‘seeds’ vs ‘seeds + spores’) and for plants growing from rhizomes in the following growing season (‘rhizomes’ vs ‘rhizomes + spores’); (2) Whether the pathogens were residing in the rhizomes during winter, causing infection in the plant the following growing season (‘rhizomes’ vs ‘infected rhizomes’); (3) Whether plants growing from rhizomes that experienced disease in the previous season were more susceptible or more resistant towards infection during the following growing season (‘rhizomes + spores’ vs ‘infected rhizomes’ vs ‘infected rhizomes + spores’). Because we used disease status of plants during collection as a proxy for infection status of the rhizome, the possibility of latent infections in rhizomes could not be excluded. Hence, healthy rhizomes were included as a control treatment to provide a baseline expectation for latent infections across treatments.

During winter, trays with rhizomes were kept in a common garden, and trays with seeds were kept in an outdoor greenhouse, covered by an agricultural polypropylene nonwoven sheet (Lutrasil, Australia) that maintained a humid soil surface to enhance seed survival. Trays with seeds and rhizomes were watered *ad libitum*. At the beginning of the following growing season (April 2020), trays with seeds were placed in the common garden and protective sheets were removed. Because the common garden (lat. 59°21′53.1″N, long. 18°03′00.9″E) was located where the plant material was collected, climatic conditions closely resembled natural conditions. Once plants started to germinate and grow, we recorded the presence and disease status of seedlings (985 seedlings in total, average 29 per tray) and plants (733 plants in total, average six per tray) throughout the growing season (April–June 2020). All observations on disease status were based on visual inspection of disease symptoms of individual ramets (García‐Guzmán & Wennström, 2001; Ericson *et al*., 2017).

### Metapopulation dynamics of pathogens

Based on the empirical estimates of spore infectivity and overwintering strategy, we set up hypotheses about the patch occupancies, colonization rates and extinction rates of each of the four pathogen species, and how these occupancies and rates would be affected by host patch size and spatial connectivity (Fig. 2). We also set up hypotheses of disease intensities within patches. Finally, we hypothesized about the effect of the spatial distribution of the alternate host, *Sorbus aucuparia*, on the occupancy pattern of Ochropsora (Fig. 2).

In order to investigate to what extent predictions derived from life‐history traits were able to explain metapopulation dynamics, we conducted an observational field study. We mapped all patches of the wood anemone within an area of *c*. 150 × 150 m, located near the sea in the Tullgarn nature reserve, Sweden (lat. 58.963683°, long. 17.612439°) (Fig. S3). Based on previous knowledge on the population structure of *A*. *nemorosa* (Shirreffs & Bell, 1984; Brunet & von Oheimb, 1998; Stehlik & Holderegger, 2000), a patch was identified as an individual patch if distanced ≥ 0.5 m from the nearest host plants (average minimum distance to the nearest patch was 2.6 m). Although it is not possible to completely rule out the possibility that patches separated by 0.5 m distance are connected via rhizomes, this distance is likely to be long enough to strongly reduce direct exchange of systemic diseases between patches (Wennström & Ericson, 1992). Yearly surveys were conducted from 2017 to 2020 at a time of the season when all diseases were easy to detect. In spring 2017, we mapped 139 patches, of which 138 could be relocated in spring 2018. During the winter of 2018–2019, about half of the study area was clear‐cut, leading to a significant loss of patches. In spring 2019 and 2020, 83 and 81 patches, respectively, were relocated. Before the clear‐cut, the area had not been disturbed by any major management activities, such as logging, for ≥ 50 yr. The loss of a few additional patches and infrequent fluctuations in patch sizes probably were caused by wild boars, which overturn large surfaces of the forest floor in search of roots and rhizomes, thereby destroying plant patches.

Every year, we measured the size (length × width; with an average ± SD of 72 ± 419 m^2^) of all patches and estimated the number of individuals (i.e. ramets) within patches. For each of the four pathogens, we recorded disease incidence (presence or absence of disease in a patch) (Fig. S4) and estimated disease intensity (proportion of diseased plants) in all patches. Following the approach of previous studies on spatial and temporal disease dynamics of rusts and smuts (Carlsson & Elmqvist, 1992; Ericson *et al*., 1999, 2017; Antonovics, 2004), disease symptoms were used as a proxy for infection status of plants. Although this method may underestimate infection occurrences and densities, it avoids destructive sampling techniques that preclude long‐term observations. To investigate whether proximity to the alternate host affects Ochropsora incidence, we also recorded the presence of *S*. *aucuparia* individuals within or near (< 1 m) the patch. Analyses based on larger distances (i.e. < 5 and < 10 m) gave similar results (results not shown).

We calculated the spatial connectivity index of the host patches (*S* ^H^) for each year (Jousimo *et al*., 2014), which represented the sum of all distances from the focal patch to all other patches (*d_ij_*) weighted by their size (*A_j_*, length × width of the patch, m^2^) and the inverse of the dispersal parameter α, in year *t*:

$$S_{\text{it}}^{H}=\sum_{j\neq i}\exp^{‐\alpha d_{\mathrm{ij}}}A_{j}$$

The inverse dispersal parameter (*α*) was set to 0.02 (50 m), based on knowledge about the annual dispersal distances of rust, chytrid and smut fungi (Alexander & Antonovics, 1988; Wennström & Ericson, 1994; Wennström & Eriksson, 1997; Piepenbring *et al*., 1998; Thrall *et al*., 2003; Powell & Letcher, 2014). We adopted the same *α*‐value for all pathogen species – thus, we assumed similar dispersal distances – to prevent biases in the results based on differences in *α*‐values (cf. Tack *et al*., 2010; Barr *et al*., 2021). Choosing an *α*‐value that was five‐fold lower or higher (0.004–0.1) did not result in qualitative changes in the results. To examine the influence of connectivity to diseased host patches (rather than the full set of host patches), we also calculated a spatial connectivity index for each pathogen (*S* ^P^) by modifying the host spatial connectivity index to only take into account diseased host patches (Methods S1) (Laine & Hanski, 2006; Jousimo *et al*., 2014). Thus, although ‘spatial connectivity of the host’ measures connectedness to all nonfocal host patches, ‘spatial connectivity of the pathogen’ measures connectedness only to diseased nonfocal host patches.

### Statistical analyses

Analyses were conducted in R v.3.6.3 (R Core Team, 2020). Bayesian models were implemented in JAGS, using the package R/rjags (Plummer, 2019). Model convergence was analyzed with the *coda.samples* function in R/coda (Plummer *et al*., 2006), and model validity and outputs were analyzed with the function *jags.samples* from R/rjags. For generalized linear models, model assumptions were validated with R/dharma (Hartig, 2020) and R/sjPlot (Lüdecke, 2020). For pathogen specific contrasts, we used the function *emmeans* of R/emmeans (Lenth, 2020). For an overview of all statistical models and technical implications, see Table S1.

#### Life‐history traits of pathogens

In order to examine the ability of spores to infect seedlings, we ran Bayesian models with the proportion of diseased seedlings in a tray (binomial distribution, logit link) modelled as a function of seed treatments (‘seeds’ and ‘seeds + spores’). To examine the ability of spores to infect plants growing from rhizomes, the capacity of pathogens to overwinter within the rhizomes, and whether rhizomes infected in the previous year were more susceptible or more resistant towards reinfection by spores in the current year, we modelled the proportion of diseased plants (i.e. ramets) in a tray (binomial distribution, logit link) as a function of the four rhizome treatments (‘rhizomes’, ‘rhizomes + spores’, ‘infected rhizomes’ and ‘infected rhizomes + spores’), separately for each pathogen. To examine whether previously infected rhizomes were more susceptible or resistant towards infection in the current year (in other words, whether the effect was nonadditive), we estimated deviations from additivity by subtracting the posterior distributions from the ‘rhizomes + spores’ and ‘infected rhizomes’ from the ‘infected rhizomes + spores’ treatment. From the posterior distributions, we calculated the range of credible values for each treatment, and the probabilities that treatments increased the proportion of infected plants or seedlings within a tray compared to healthy controls. Plants in healthy control treatments, including ‘seeds’ as well as ‘rhizomes’, never showed any signs of disease. Because we were interested in the effects of quantitative trait differences rather than qualitative differences based on a strict cut‐off value, we opted for the framework of Bayesian statistics to analyze pathogen traits (Gelman *et al*., 1995).

#### Metapopulation dynamics of pathogens

In order to examine whether pathogens differed in their patch occupancy and colonization and extinction rates, we modelled patch occupancy, colonization rate and extinction rate as functions of pathogen species identity using generalized linear mixed‐effects models (GLMM) with binomial distributions. When modelling patch occupancy, the response variable was incidence of disease (0 or 1) in host patches. When modelling colonization rates, the response variable represented all host patches that did not show disease in year *t*−1, which either remained without disease (0) or became colonized (1) in year *t*. When modelling extinction rates, the response variable represented all host patches that were diseased in year *t*−1, which either remained diseased (0) or were without disease (1) in year *t*. Year was included as a random effect in these models to take into account temporal variation.

In order to analyze the effect of the distribution of the host on patch occupancy, we modelled the effect of host patch size (log_e_‐transformed), spatial connectivity of the host patches (*S* ^H^, log_e_‐transformed) and their interaction on patch occupancy of each pathogen during each year (i.e. pathogen‐ and year‐specific models), using generalized linear models with binomial distribution. To analyze the effect of host and pathogen distributions on colonization and extinction rates, we modelled the effect of host patch size (log_e_‐transformed), spatial connectivity of the pathogen (*S* ^P^, log_e_‐transformed) and their interaction on the colonization and extinction rates of each pathogen across all years (i.e. pathogen‐specific models), using GLMM with binomial distributions. Because we expected conditions in the preceding year to affect recorded colonizations and extinctions in the following year, we used pathogen spatial connectivity and host patch size from year *t*−1 in our models. To account for temporal variation in colonization and extinction rates, we included yearly transition (i.e. 2017 to 2018, 2018 to 2019 or 2019 to 2020) as a random effect in these models.

In order to examine whether disease intensities within host patches differed among pathogen species, we modelled the average proportion of diseased plants within a patch as a function of pathogen species. To account for temporal variation in disease intensities, year was included as a random effect. Host patch size (scaled to mean zero and unit variance) was included as a covariate in the model.

In order to investigate the spatial relationship between Ochropsora and the alternate host of Ochropsora, we modelled the effect of presence of *S*. *aucuparia* on patch occupancy by Ochropsora. To account for temporal variation in patch occupancy by Ochropsora, year was included as a random effect. To take into account the influence of host patch size on Ochropsora occupancy and *S*. *aucuparia* presence, we included host patch size (scaled to mean zero and unit variance) as a covariate in these models.

## Results

### Life‐history traits of pathogens

**Fig. 1:**
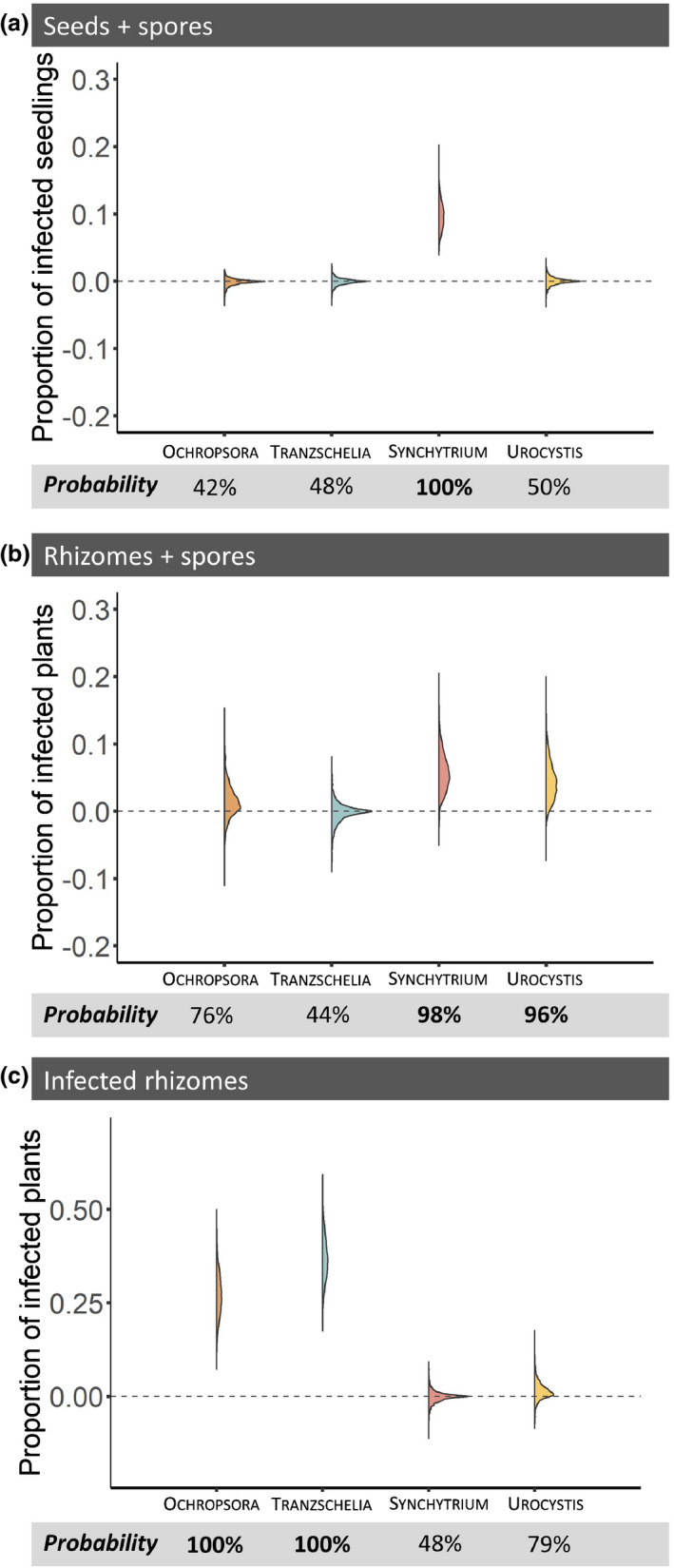
Half‐violin plots of the posterior probability distributions, which visualize the probabilities for proportions of infected plants within trays for Ochropsora (orange), Tranzschelia (blue), Synchytrium (red) and Urocystis (yellow) when (a) seedlings were inoculated with spores, (b) rhizomes were inoculated with spores and (c) rhizomes were infected previously. Below each panel, the probability that a treatment will have more infected plants than the control treatment is given (i.e. the proportion of the probability distribution that is above zero). Probabilities > 95% are shown in bold. See Supporting Information Fig. S2 for results on the nonadditive effect of spore inoculation and previous infection on the proportion of infected plants. See Supporting Information Table S1 for details of the fitted models.

Seedlings inoculated with Synchytrium spores showed disease in 10% of the cases, whereas spores of the other pathogen caused no visible disease symptoms (Fig. 1a; Table S2). Plants growing from rhizomes inoculated with Synchytrium or Urocystis spores had probabilities of 6% and 4%, respectively, of showing disease, whereas plants growing from rhizomes inoculated with Ochropsora spores only had a 1% probability of being diseased, and inoculation with Tranzschelia spores caused no disease (Fig. 1b; Table S2). Plants growing from Ochropsora‐ and Tranzschelia‐infected rhizomes without spore inoculation had probabilities of 27% and 37%, respectively, of being diseased, whereas plants growing from Synchytrium‐ or Urocystis‐infected rhizomes had 0% and 1% probability of being diseased, respectively (Fig. 1c; Table S2). Thus, although Synchytrium and Urocystis had a higher probability of infecting plants via their spores, Ochropsora and Tranzschelia had a higher probability of overwintering in the rhizomes through to the next growing season (Table S2).

Plants shooting from Tranzschelia‐, Synchytrium‐ or Urocystis‐infected rhizomes were neither more susceptible nor more resistant towards reinfection by Tranzschelia, Synchytrium or Urocystis spores, respectively (Fig. S2; Table S2). However, plants growing from Ochropsora‐infected rhizomes were more susceptible to reinfection: upon inoculation with spores, plants from infected rhizomes had a 12% higher probability of showing disease than healthy rhizomes (Fig. S2; Table S2).

### Metapopulation dynamics of pathogens

**Fig. 2:**
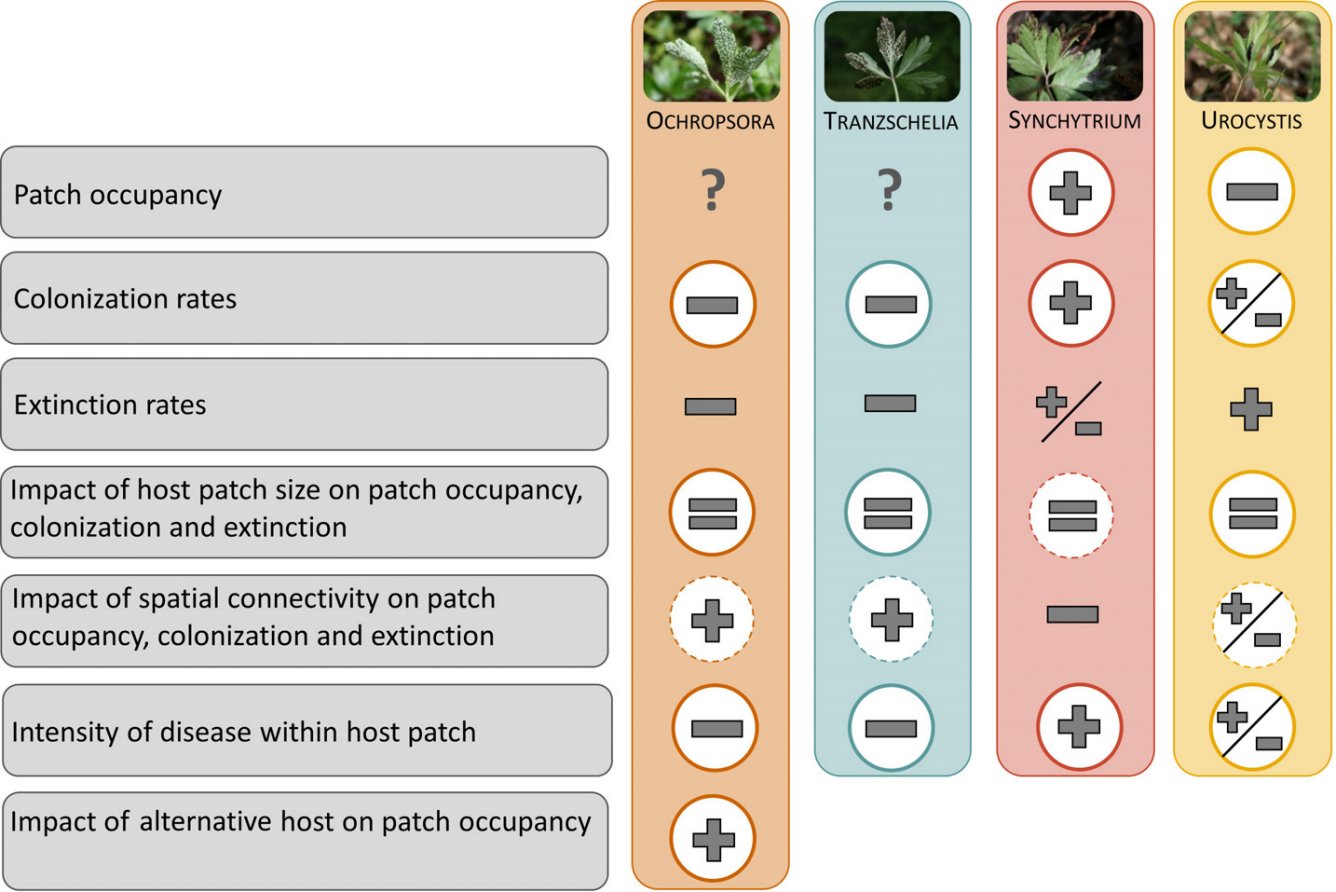
Schematic overview of our hypotheses on the spatial and temporal dynamics of the pathogens Ochropsora, Tranzschelia, Synchytrium and Urocystis, as based on their life‐history traits. Symbols represent our predictions, and indicate low (−), medium (+/−) or high (+) predicted values or impacts. Impacts predicted to be similar for all pathogens are indicated with a ‘=’, and values or impacts for which we had no information to base predictions on are indicated with a ‘?’. For example, for patch occupancy, we had no *a priori* prediction for Ochropsora and Tranzschelia, whereas we predicted high patch occupancy for Synchytrium, and low patch occupancy for Urocystis. Because we expected the effect of host patch size on disease dynamics to be independent of pathogen traits, we predicted similar impacts (=) for all pathogens. All circled predictions were confirmed by our data, with strong support for predictions indicated by thick and solid circles, and weak support for predictions indicated by thin and dashed circles. For a complete overview of all predictions, hypotheses and results, see Supporting Information Table S3. In brief, pathogens that relied mostly on systemic infection of rhizomes and less on spore dispersal (Ochropsora, Tranzschelia) were expected to have low colonization and extinction rates, leading to unpredictable patch occupancies. Moreover, we expected these pathogens, relying mostly on rhizome infection, to be highly dependent on spatial connectivity, and have a restricted distribution within patches. Occupancy of Ochropsora was expected to be dependent on the presence of the alternate host. For the two pathogens that relied mostly on spore dispersal, we expected high colonization rates for Synchytrium, as a result of its ability to infect both adult plants and seedlings via spores, and intermediate colonization rates for Urocystis, as a result of its ability to infect adult plants via spores. We expected the extinction rates of these two species to be high due to low or no ability to overwinter within rhizomes. By contrast, we expected the incidence of Synchytrium and Urocystis in patches to be relatively independent on spatial connectivity, and to have an efficient spread of disease within patches. Lastly, we expected that patch occupancy, and colonization and extinction rates of all pathogens were influenced by host patch size.

**Fig. 3:**
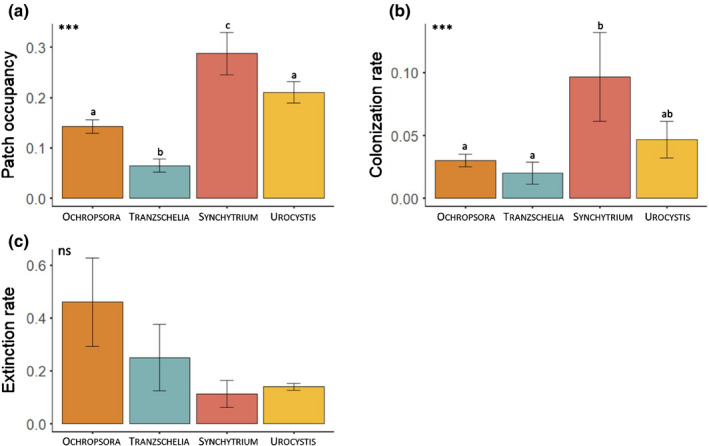
Metapopulation dynamics of Ochropsora (orange), Tranzschelia (blue), Synchytrium (red) and Urocystis (yellow). Panels show (a) patch occupancy, (b) colonization rates and (c) extinction rates, averaged across all years with error bars showing SD. Stars indicate significance of the model (***, *P* < 0.001; ns, nonsignificant), and letters indicate pairwise differences between pathogen species. For fitted models, see Supporting Information Table S1, and for *P*‐values and test statistics of the models and pathogen‐specific contrasts, see Table S7. Table S4 presents values of patch occupancy, colonization and extinction rates for each pathogen during each of the four years. For year‐specific patch occupancies for each of the four pathogens, see Fig. S5(a).

Based on the quantitative estimates of life‐history traits, we set up hypotheses regarding the metapopulation dynamics of each of the four pathogens, as presented in Fig. 2 and Table S3. As hypothesized based on traits, Synchytrium had a significantly higher patch occupancy than the other pathogens (Fig. 3a; Table S5). Ochropsora and Urocystis had intermediate patch occupancies, whereas Tranzschelia had significantly lower patch occupancies than all other pathogens (Fig. 3a; Table S5). Colonization rate was highest for Synchytrium, intermediate for Urocystis, and lowest for Ochropsora and Tranzschelia, partly confirming our hypotheses (Fig. 3b; Table S5). Extinction rates did not differ significantly among pathogen species, and were highly variable among years, especially so for Ochropsora (8–45%) and Tranzschelia (0–50%), and less so for Synchytrium (0–20%) and Urocystis (11–16%) (Fig. 3c; Table S5).

**Fig. 4:**
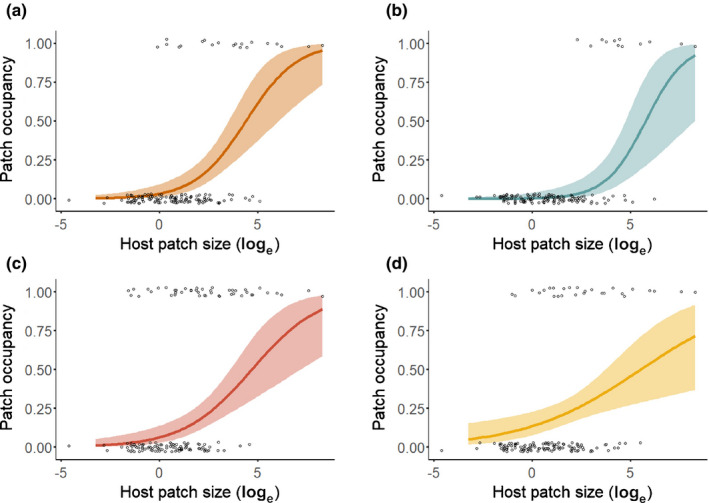
The effect of host patch size (log_e_‐transformed) on patch occupancy of (a) Ochropsora, (b) Tranzschelia, (c) Synchytrium and (d) Urocystis during 2018, including the full set of host patches (*n* = 138). Lines represent the predicted relationship with 95% confidence interval. All relationships were significant (*P* < 0.001). For fitted models, see Supporting Information Table S1, and for *P*‐values and test statistics of the models see Table S6.

**Fig. 5:**
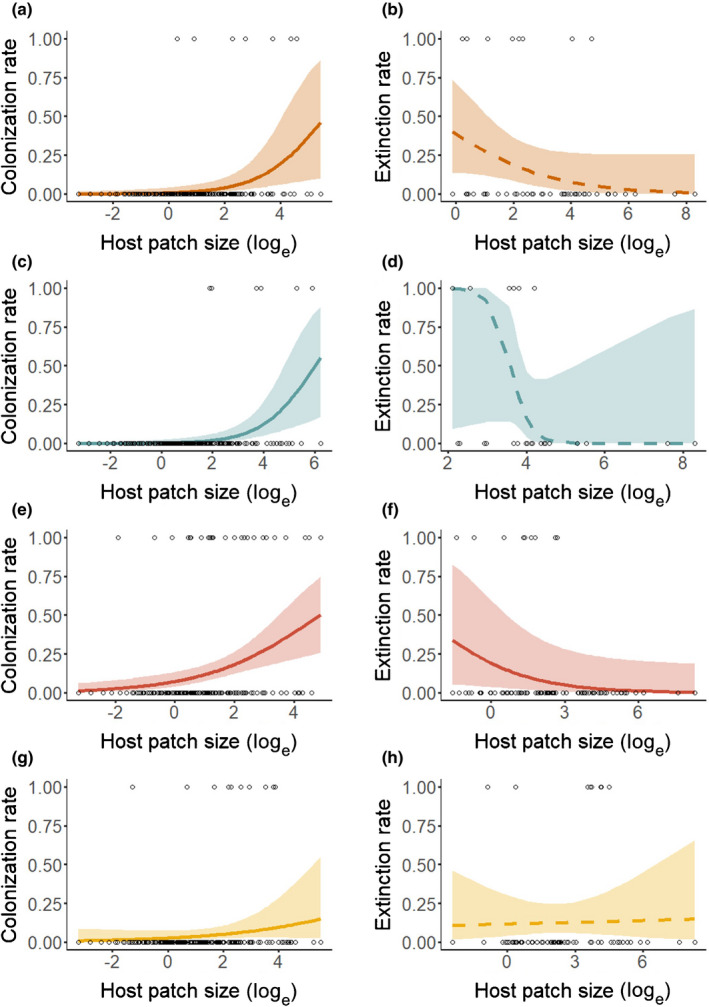
The effect of host patch size (log_e_‐transformed) on colonization (left) and extinction rates (right), for (a, b) Ochropsora, (c, d) Tranzschelia, (e, f) Synchytrium and (g, h) Urocystis, during all survey years (2017–2020). For colonization rate, the *y*‐axis represents the proportion of unoccupied patches in year *t*−1 that became occupied in year *t*. For extinction rate, the y‐axis represents the proportion of occupied patches in year *t*−1 that became unoccupied in year *t*. Note that for panels on colonization rates, only patches that were unoccupied in year *t*−1 are included, whereas for panels on extinction rates, only patches that were occupied in year *t*−1 are included. Lines represent the predicted relationship with 95% confidence interval. Significant relationships have solid lines (*P* < 0.05), and nonsignificant relationships dashed lines. For fitted models, see Supporting Information Table S1, and for *P*‐values and test statistics of the models see Table S7.

**Fig. 6:**
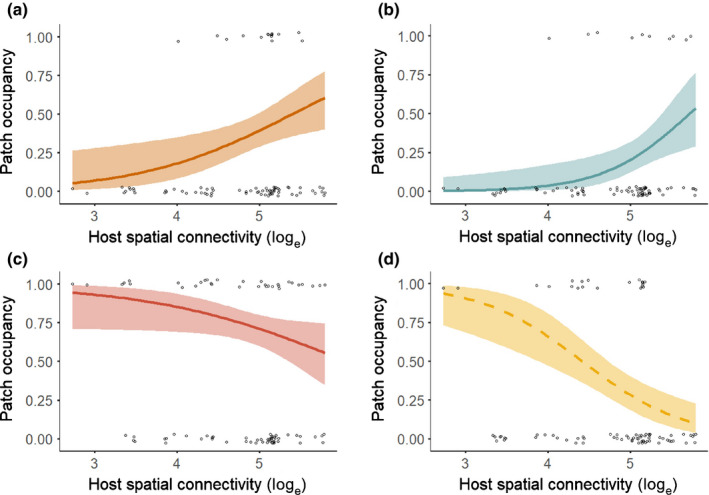
The effect of host spatial connectivity (log_e_‐transformed) on patch occupancy by (a) Ochropsora, (b) Tranzschelia, (c) Synchytrium and (d) Urocystis during 2019. Host spatial connectivity accounts for the distance from the focal patch to all other patches, as well as the size of all nonfocal patches. Lines represent the predicted relationship with 95% confidence interval. Solid lines represent significant relationships (*P* < 0.05), dashed lines represent trends (*P* < 0.1). For fitted models, see Supporting Information Table S1, and for *P*‐values and test statistics of the models see Table S6. See Fig. S6 for the relationship between host spatial connectivity and patch occupancy separately for each survey year.

**Fig. 7:**
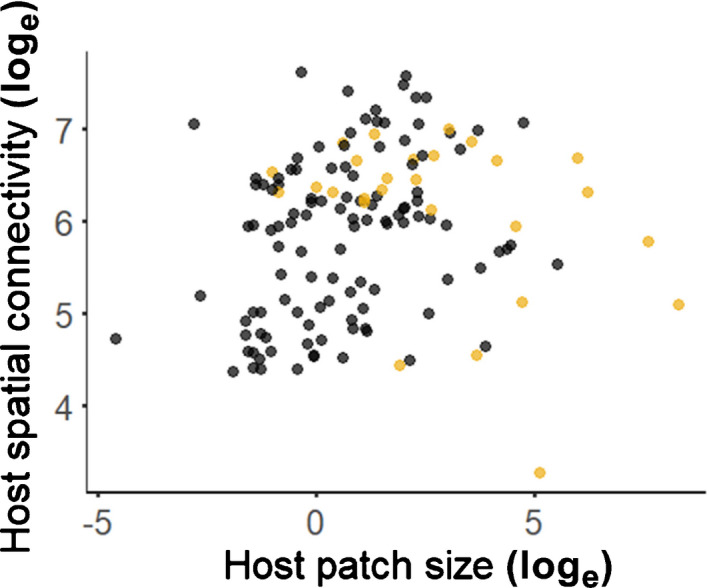
Interactive effect of host patch size and host spatial connectivity (log_e_‐transformed) on incidence of Urocystis in 2018. Host spatial connectivity accounts for the distance from the focal patch to all other patches, as well as the size of all patches. Yellow dots represent patches occupied by Urocystis, black dots represent patches not occupied by Urocystis. For small host patches, Urocystis is present mostly when host patches have a high connectivity, whereas larger host patches are diseased also when connectivity is low. For a visualization of the interactive effects for 2017 and 2019, see Supporting Information Fig. S7. For fitted models, see Table S1, and for *P*‐values and test statistics of the models see Table S6.

As hypothesized, host patch size was positively related to patch occupancy for all four pathogens during all four years (Fig. 4; Table S6). Host patch size also was positively related to colonization rate for all pathogens (Fig. 5; Table S7). Synchytrium had fewer extinctions in larger patches, but, contrary to our hypothesis, the extinction rates of Ochropsora, Tranzschelia and Urocystis were not significantly related to host patch size (Fig. 5; Table S7). Host spatial connectivity was positively related to Ochropsora and Tranzschelia patch occupancy in two of four years, which supported our hypothesis, whereas Synchytrium patch occupancy was negatively related to host spatial connectivity in three out of four years (Figs 6, S6; Table S6). The effect of host spatial connectivity on Urocystis patch occupancy was dependent on host patch size (Table S6), with smaller patches being positively related to host spatial connectivity, but not larger patches (Figs 7, S7). Pathogen spatial connectivity was not related to the colonization and extinction rates of any of the pathogens (Fig. S8; Table S7).

The disease intensity within a patch differed significantly among pathogens ($\chi_{3}^{2}$ = 276.77, *P* < 0.001). Synchytrium had the highest disease intensity within patches during all years (20.5% of plants diseased on average), Urocystis had intermediate disease intensity (3.1%), and Ochropsora and Tranzschelia had the lowest disease intensity (0.6% and 0.4% respectively), which supported our hypothesis (Fig. S5b; Table S8).

As hypothesized, patches in close proximity to *S*. *aucuparia* (≤ 1 m) had a higher probability of being diseased by Ochropsora than patches without *S*. *aucuparia* nearby (38% vs 9%, respectively; $\chi_{1}^{2}$ = 24.34, *P* < 0.001) (Fig. S9).

## Discussion

Our study explored the interplay between life‐history traits and metapopulation dynamics of four fungal pathogens on *Anemone nemorosa*, by combining experiments, theory and field surveys into a unified framework. The two rust fungi (Ochropsora and Tranzschelia) had a higher probability of causing perennial systemic infections, whereas the smut (Urocystis) and chytrid fungus (Synchytrium) had a higher probability of infecting plants via spores. Synchytrium was the only pathogen that could infect seedlings. Our hypotheses on the relationships between traits and disease dynamics were partly confirmed (Fig. 2). Pathogens that had a higher probability to cause perennial systemic infections also had lower patch occupancies, lower colonization rates and lower within‐patch disease levels than pathogens that had a higher probability to infect through spores. As hypothesized, patch occupancy of Ochropsora was strongly related to the presence of its alternate host. As expected, the effect of host patch size on occupancy and colonization rates was positive for all pathogen species, irrespective of traits. Contrary to expectations based on traits, extinction rates did not differ among the pathogen species, and spatial connectivity did not consistently affect patch occupancy and colonization and extinction rates. The approach adopted in our study – using experimental assays to identify and quantify life‐history traits of different pathogen species attacking the same host, and then use metapopulation theory to formulate hypotheses that are tested by field surveys – provides an important tool to examine how and when traits influence metapopulation dynamics.

### Link between traits, and patch occupancy, colonization rate and extinction rate

Our findings largely supported our hypotheses on how life‐history traits were expected to be related to patch occupancies and colonization rates of the pathogens (Fig. 2). The fact that pathogens that had higher probabilities to infect plants and seedlings (Synchytrium) or plants (Urocystis) via spores had higher patch occupancies and colonization rates than the species that had a higher probability to cause perennial systemic infections (Ochropsora and Tranzschelia) indicates that colonization of new host patches is more likely to happen in pathogen species that infect new hosts mainly via spores, particularly in species where spores can infect both seedlings and adult plants, and that colonization events are less likely when pathogens mainly rely on rhizomatous spread of disease. Unfortunately, few studies have focused on pathogens that have the ability to transmit systemically, and comparative studies on the differences in metapopulation dynamics of pathogens with distinct life histories sharing a single host are lacking. However, previous studies on pathogen species that mainly rely on transmission via spores found that these species are characterized by frequent colonizations, although reported patch occupancies vary among studies (Burdon *et al*., 1995; Ericson *et al*., 1999, 2017; Antonovics, 2004; Laine & Hanski, 2006; Smith *et al*., 2011). For example, although patch occupancy of the powdery mildew *Podosphaera plantaginis* on *Plantago lanceolata* patches ranged from *c*. 1% to *c*. 5% during four years, patch occupancy ranged from *c*. 10% to *c*. 35% during a 14‐yr period for the smut fungus *Ustilago violacea* on *Silene latifolia* (Antonovics, 2004), and from *c*. 40% to *c*. 100% during a 28‐yr period for the rust fungus *Uromyces valerianae* on *Valeriana salina* (Ericson *et al*., 2017). Although the pathogen species investigated in this study differed strongly in life‐history traits, it would be interesting also to investigate if more modest variation in single traits can explain quantitative differences in disease dynamics, for example by studying a larger set of pathogens or strains with the same transmission mode (Tack *et al*., 2014; Burdon & Laine, 2019). Also, in order to elucidate whether transmission mode has important effects on metapopulation dynamics, we need field experiments, where incidence of infection is recorded after spore exclusion and inoculation, or after introduction of sentinel plants.

The observed differences in patch occupancies of the four pathogens are likely to be driven mainly by colonization rates, as there were no differences in extinction rates among pathogens. We did not detect any consistent patterns in extinction rates of pathogens among years, but extinction rates of Ochropsora and Tranzschelia were remarkably high (i.e. 45% and 50%, respectively) in some years. A possible explanation for this is that Ochropsora and Tranzschelia had low disease intensities within patches (i.e. small population sizes), and these pathogens could thus be more prone to go extinct as a result of demographic and environmental stochasticity, or of recovery of a few host plants (Antonovics, 2004). It also is important to consider the possibility that some of the observed extinctions of the perennial systemic pathogens may not be true extinctions, as infection can remain latent within the rhizomes without causing visible symptoms in the host (Wennström & Ericson, 1994). We did indeed observe a few instances where symptoms of Ochropsora infection reappeared after one or two years of absence from the patch, which could be a consequence either of latency or re‐colonization, although these instances were too few to analyze formally. In order to uncover the frequency of latency, future studies could track the disease status of individual plants within and among patches during multiple years, and use molecular tools to verify latency.

### The effect of host patch size and spatial connectivity on metapopulation dynamics

In line with metapopulation theory, larger patches had higher occupancies and higher colonization rates, irrespective of pathogen life‐history traits. Although we also expected fewer extinctions in larger patches for all pathogens, this relationship was found only for Synchytrium. Higher occupancy, higher colonization rates and lower extinction rates in larger patches all are central predictions of metapopulation theory, yet our results with no effects on extinction rates match empirical studies on other wild plant pathogens: these studies often report a positive effect of host patch size on occupancy and colonization (Jennersten *et al*., 1983; Carlsson & Elmqvist, 1992; Burdon *et al*., 1995; Ericson *et al*., 1999; Smith *et al*., 2003; Laine & Hanski, 2006), but no effect of host patch size on extinction (Laine & Hanski, 2006).

Host spatial connectivity was positively related to patch occupancy of the two perennial systemic pathogens (Ochropsora and Tranzschelia), which was in line with our hypothesis (Fig. 2). Although comparable studies on perennial systemic pathogens are scarce, these results are in line with a previous study on plant metapopulation dynamics by Dupré & Ehrlén (2002), who found that clonal perennial plants are more negatively affected by isolation than annual plants with seed production, likely as a consequence of a lower dispersal ability. Regarding pathogens infecting mainly via spores, we found that the effect of host spatial connectivity on Urocystis occupancy was dependent on host patch size, that is, isolation negatively affected occupancy of small but not large patches. Synchytrium was able to infect isolated patches also, and, surprisingly, this pathogen species was more likely to occupy isolated patches than connected patches. It is possible that the environmental conditions of some of the isolated patches were particularly favourable for Synchytrium. For example, some patches close to the sea were isolated but growing under extremely moist conditions, potentially promoting flagellar movement and germination success of the zoospores (Powell & Letcher, 2014). Besides such direct effects of the environment, local environmental conditions also may indirectly influence pathogen infection and disease development via impacts on host phenotype, such as host susceptibility (Anacker *et al*., 2008). Although most metapopulation models do not incorporate the direct and indirect effects of local environmental conditions, this can indeed be an important determinant for population dynamics (Moilanen & Hanski, 1998; Harrison *et al*., 2011). We did not find a positive relationship between host spatial connectivity and the presence of Synchytrium and Urocystis at the spatial scale investigated (*c*. 2 ha), yet it is still possible that the distributions of Synchytrium and Urocystis at larger spatial scales are limited by dispersal. Indeed, studies that have focused on larger spatial scales (spanning tens of kilometres) have found positive effects of spatial connectivity on disease incidences of pathogens with wind‐dispersed spores (Burdon *et al*., 1995; Laine & Hanski, 2006; Carlsson‐Granér *et al*., 2014). Our study focused on the metapopulation dynamics in a limited area, and it cannot be excluded that processes occurring over larger spatial scales might have had some influence on the dynamics observed in our study area. In contrast to the effects on patch occupancies, pathogen spatial connectivity did not affect colonization and extinction rates in any of the species. This result was unexpected based on theoretical predictions (Hanski, 1994) and findings of previous studies (Thrall *et al*., 2003; Laine & Hanski, 2006). Laine & Hanski (2006) found that colonization of new *P. lanceolata* patches by the powdery mildew fungus *P. plantaginis* was more likely when other infected patches were nearby, and in some years, well‐connected patches were more likely to have persistent infection, suggesting rescue effects. To untangle the independent and joint effects of pathogen traits and spatial scale on the relationship between spatial connectivity and colonization and extinction dynamics, future studies might focus on pathogens with distinct life histories across multiple spatial scales.

### Link between traits and within‐patch disease intensities

The pathogen species infecting both adult plants and seedlings (Synchytrium) had several‐fold higher disease intensities within patches than the other pathogen species, supporting our hypothesis that it is able to spread efficiently throughout a colonized patch. This pattern may be reinforced by the ability of its zoospores to move through water films (Powell & Letcher, 2014). Urocystis, which also infects new hosts via spores but is unable to infect seedlings, had an intermediate disease intensity within occupied patches, whereas the perennial systemic rusts Ochropsora and Tranzschelia had lower disease intensities. These findings suggest that, as expected, dispersal via spores is more efficient than systemic dispersal via rhizomes, also within patches. In line with these findings, a previous study on a perennial systemic pathogen (the rust fungus *Puccinia minussensis* on *Lactuca sibirica*) found that spread of disease through rhizomes was strongly limited by distance; plants that shared a rhizome with an infected plant could escape infection completely when growing at a distance > 45 cm (Wennström & Ericson, 1992). Besides the influence of pathogen traits on short‐distance dispersal, disease intensity within patches also might be affected by competition among pathogens (Strauss *et al*., 2019). Conducting a meta‐analysis across study systems would be one way to further explore general trends in the link between transmission mode and within‐patch disease intensities. As disease intensities may, in turn, affect survival of seeds, seedlings and plants (Burdon, 1987), such insights could not only be useful in describing disease dynamics, but also shed light on the consequences for population dynamics of the host plant.

### The effect of spatial distribution of the alternate host on metapopulation dynamics

Because one of the studied pathogens (Ochropsora) needs two hosts to complete its sexual life cycle, we expected that presence of the alternate host would have a strong effect on the metapopulation dynamics of Ochropsora. We found that patches were indeed more likely to be infected by Ochropsora when growing close to *S*. *aucuparia*, as also has been shown in a previous study on this pathogen (García‐Guzmán & Wennström, 2001). This effect of the alternative host signifies the importance of considering the full life cycle of an organism when aiming to describe its distribution (Burdon & Thrall, 2014). Host alternation has been identified as an important determinant for species distributions of other taxa, such as insects (Yukawa *et al*., 2007; Bell *et al*., 2012) and trematodes (Jokela & Lively, 1995). For pathogens with sessile hosts, such as plants, a strong correlation in space between the two hosts and pathogen incidence should be expected (Jacobi *et al*., 1993; Farkas *et al*., 2016). A striking example of such a correlation stems from the beginning of the 20^th^ Century, when the United States initiated a massive eradication program of barberry (*Berberis vulgaris*), the alternate host of the wheat stem rust *Puccinia graminis*, to protect wheat crops from this disease. Ever since these eradication efforts, epidemics of wheat stem rust have sharply declined in all managed areas (Leonard, 2001). This case, and the results of our study, exemplify the importance of acknowledging the full life cycle of a pathogen when designing methods to prevent disease outbreaks in natural or agricultural systems.

### Conclusion

Our study introduces a promising approach for investigating the relationships between pathogen life‐history traits and metapopulation dynamics, by comparing multiple pathogen species sharing the same host plant and combining experimental assessments of life‐history traits and metapopulation theory with extensive field surveys of spatial population dynamics. Using this integrative approach, we were able to show that although some aspects of pathogen metapopulation dynamics were related to life‐history traits in the predicted way, other aspects appeared to be less predictable from the life‐history traits investigated here. Most likely, acquiring knowledge on additional life‐history traits of both pathogen and host plant, and taking into account the direct and indirect effects of environmental factors on pathogens and host phenotype (Stevens, 1960) will improve the explanatory power of this approach. Gaining understanding of the influence of pathogen life histories on how, when and where pathogens interact with their hosts contributes to our fundamental knowledge on disease ecology and coevolutionary dynamics between pathogen and host in natural systems. From an applied perspective, we could adopt this integrated approach to improve our ability to predict host–pathogen dynamics, which is of vital importance to prevent disease invasions and disease outbreaks, as it helps to identify the need for quarantining and predict the efficacy of biological control.

## Author contributions

LJAvD, AJMT and JE designed the research; and LJAvD conducted the field‐ and experimental work, analyzed the data and wrote the manuscript, with contributions from all authors.

## Supporting information


**Fig. S1** Pathogen treatments of seeds and rhizomes in the common garden, including ‘seeds’, ‘seeds + spores’, ‘rhizomes’, ‘rhizomes + spores’, ‘infected rhizomes’ and ‘infected rhizomes + spores’.
**Fig. S2** Half‐violin plots of the posterior probability distributions, which visualize the nonadditive effect of spore inoculation and previous infection on the proportion of diseased plants within trays for Ochropsora, Tranzschelia, Synchytrium and Urocystis.
**Fig. S3** Map of all 139 patches of *Anemone nemorosa* in the study area within the Tullgarn nature reserve, 2017.
**Fig. S4** Disease status of all patches of *Anemone nemorosa* for Ochropsora, Tranzschelia, Synchytrium and Urocystis in the study area within the Tullgarn nature reserve, 2018.
**Fig. S5** Patch occupancy and disease intensities within host patches in 2017, 2018, 2019 and 2020.
**Fig. S6** Effect of host spatial connectivity (log_e_‐transformed) on patch occupancy for Ochropsora, Tranzschelia, Synchytrium and Urocystis in each of the survey years.
**Fig. S7** Interactive effect of host patch size (log_e_‐transformed) and host spatial connectivity (log_e_‐transformed) on incidence of Urocystis in 2017, 2018 and 2019.
**Fig. S8** Effect of pathogen spatial connectivity (log_e_‐transformed) on colonization and extinction rates for Ochropsora, Tranzschelia, Synchytrium and Urocystis, during all survey years (2017–2020).
**Fig. S9** Relationship between presence of *Sorbus aucuparia* (≤ 1 m from the host patch) and patch occupancy by Ochropsora.
**Methods S1** Calculation of the spatial connectivity indices.
**Table S1** Overview of fitted models to examine life‐history traits of pathogens and their metapopulation dynamics.
**Table S2** Effects of treatments on the proportion of diseased plants, and the probability that rhizomes inoculated with spores have a higher proportion of disease than previously infected rhizomes, separately for each pathogen.
**Table S3** Overview of our hypotheses on the spatial and temporal dynamics of Ochropsora, Tranzschelia, Synchytrium and Urocystis pathogens, as based on the life‐history traits of each pathogen.
**Table S4** Metapopulation dynamics of Ochropsora, Tranzschelia, Synchytrium and Urocystis pathogens from 2017 to 2020, including the number of diseased host patches, patch occupancy, colonization and extinction events and rates, and the disease intensity within a host patch.
**Table S5** Effect of pathogen identity on path occupancy and colonization and extinction rates, and the results of pathogen‐specific contrasts of the significant models, using the function *emmeans* in R/emmeans.
**Table S6** Effect of host patch size, host spatial connectivity and their interaction on patch occupancy.
**Table S7** Effects of host patch size, spatial connectivity of the pathogen, and their interaction on colonization and extinction rates of Ochropsora, Tranzschelia, Synchytrium and Urocystis, from 2017 to 2020.
**Table S8** Effect of pathogen identity on disease intensity within a host patch.
Please note: Wiley Blackwell are not responsible for the content or functionality of any Supporting Information supplied by the authors. Any queries (other than missing material) should be directed to the *New Phytologist* Central Office.

## Data Availability

Data available from the Dryad Digital Repository: https://doi.org/10.5061/dryad.5qfttdz6z
