# Supplementary material for: The relationship between pathogen life‐history traits and metapopulation dynamics
Source: New Phytol. 2022 Jan 22;233(6):2585–98. doi: 10.1111/nph.17948 (PMC9306763; doi:10.1111/nph.17948)
Supplement: Supplementary file 1 — Fig. S1 Pathogen treatments of seeds and rhizomes in the common garden, including ‘seeds’, ‘seeds + spores’, ‘rhizomes’, ‘rhizomes + spores’, ‘infected rhizomes’ and ‘infected rhizomes + spores’. Fig. S2 Half‐violin plots of the posterior probability distributions, which visualize the nonadditive effect of spore inoculation and previous infection on the proportion of diseased plants within trays for Ochropsora, Tranzschelia, Synchytrium and Urocystis. Fig. S3 Map of all 139 patches of Anemone nemorosa in the study area within the Tullgarn nature reserve, 2017. Fig. S4 Disease status of all patches of Anemone nemorosa for Ochropsora, Tranzschelia, Synchytrium and Urocystis in the study area within the Tullgarn nature reserve, 2018. Fig. S5 Patch occupancy and disease intensities within host patches in 2017, 2018, 2019 and 2020. Fig. S6 Effect of host spatial connectivity (loge‐transformed) on patch occupancy for Ochropsora, Tranzschelia, Synchytrium and Urocystis in each of the survey years. Fig. S7 Interactive effect of host patch size (loge‐transformed) and host spatial connectivity (loge‐transformed) on incidence of Urocystis in 2017, 2018 and 2019. Fig. S8 Effect of pathogen spatial connectivity (loge‐transformed) on colonization and extinction rates for Ochropsora, Tranzschelia, Synchytrium and Urocystis, during all survey years (2017–2020). Fig. S9 Relationship between presence of Sorbus aucuparia (≤ 1 m from the host patch) and patch occupancy by Ochropsora. Methods S1 Calculation of the spatial connectivity indices. Table S1 Overview of fitted models to examine life‐history traits of pathogens and their metapopulation dynamics. Table S2 Effects of treatments on the proportion of diseased plants, and the probability that rhizomes inoculated with spores have a higher proportion of disease than previously infected rhizomes, separately for each pathogen. Table S3 Overview of our hypotheses on the spatial and temporal dynamics of Ochropsora, Tranzschelia, Synchytr [file NPH-233-2585-s001.pdf]

New Phytologist Supporting Information

Article title: The relationship between pathogen life history traits and metapopulation dynamics

Authors: Laura J. A. van Dijk, Johan Ehrlén and Ayco J. M. Tack

Article acceptance date: 17 December 2021

Table S1. Overview of fitted models to examine life history traits of pathogens and their metapopulation dynamics. For pathogen traits, models analysed the effect of spore inoculation and previous rhizome infection on the proportion of infected plants. For metapopulation dynamics, models analysed differences among pathogens in terms of patch occupancy and colonization and extinction rates, the effect of spatial connectivity and host patch size on these occupancies and rates, and differences among pathogens in terms of disease intensities within patches. β represents slopes, [R] indicates random effects, S^H = spatial connectivity of the host, S^P = spatial connectivity of the pathogen, A = host patch size (length × width).

**Question**	**Response variable**	**Predictor variables**	**Fitted models**	**Model type and distribution**
What is the effect of spore inoculation on the proportion of diseased seedlings?	Proportion of diseased seedlings within a tray	β_1 × "seeds" +<br>β_2 × "seeds + OA spores" +<br>β_3 × "seeds + TA spores" +<br>β_4 × "seeds + SA spores" +<br>β_5 × "seeds + UA spores"	All pathogens included in 1 model	Bayesian, binomial
What is the effect of spore inoculation and previous rhizome infection on the proportion of diseased plants?	Proportion of diseased plants within a tray[1]	β_1 × "rhizomes" +<br>β_2 × "rhizomes + spores" +<br>β_3 × "infected rhizomes" +<br>β_4 × "infected rhizomes + spores"	Pathogen-specific (4 models)	Bayesian, binomial

[1] Disease status was observed for each individual ramet

Do pathogens differ in patch occupancies?	Patch occupancy	~ Pathogen identity + Year [R]	All pathogens and years included in 1 model	Glmer, binomial
Do pathogens differ in colonization rates?	Colonized vs. non-colonized patches (1/0) during each year	~ Pathogen identity + Year [R]	All pathogens and years included in 1 model	Glmer, binomial
Do pathogens differ in extinction rates?	Extinct vs. persistent pathogen populations (1/0) during each year	~ Pathogen identity + Year [R]	All pathogens and years included in 1 model	Glmer, binomial
What is the effect of host patch size and spatial connectivity of the host on patch occupancy in one year?	Patch occupancy	$\sim \log(S^H) + \log(A) + \log(S^H) \times \log(A)$	Pathogen- and year-specific (16 models)	Glm, binomial
What is the effect of host patch size and spatial connectivity of the pathogen on colonization rates?	Colonized vs. non-colonized patches (1/0) during each year	$\sim \log(S^P_{t-1}) + \log(A_{t-1}) + \log(S^P_{t-1}) \times \log(A_{t-1})$ + Year [R]	Pathogen-specific (4 models)	Glmer, binomial
What is the effect of host patch size and spatial connectivity of the pathogen on extinction rates?	Extinct vs. persistent pathogen populations (1/0) during each year	$\sim \log(S^P_{t-1}) + \log(A_{t-1}) + \log(S^P_{t-1}) \times \log(A_{t-1})$ + Year [R]	Pathogen-specific (4 models)	Glmer, binomial

Do pathogens differ in disease intensity within a host patch?	disease intensity (ln-transformed)	~ Pathogen identity + Host patch size (scaled) + Year [R]	All pathogens and years included in 1 model	Lmer, Gaussian
Does the presence of the alternate host affect patch occupancy by Ochropsora?	Ochropsora patch occupancy	~ Presence of alternate host + Host patch size (scaled) + Year [R]	Pathogen-specific, only on Ochropsora, 1 model	Glmer, binomial

Table S2. **(a)** Effects of treatments (“seeds + spores”, “rhizomes + spores”, “infected rhizomes” and “infected rhizomes + spores”) on the proportion of diseased plants. Shown are the mean proportions of diseased plants within each treatment, with 95% credible intervals in parentheses. See figs. 1 and S2 for posterior probability distributions. **(b)** The probability that rhizomes inoculated with spores have a higher proportion of disease than previously infected rhizomes, separately for each pathogen, with the mean values and 95% credible intervals. Probabilities close to one indicate that spore inoculation is much more probable to cause disease than previously infected rhizomes (accompanied by positive mean values). Probabilities close to zero indicate that infected rhizomes are much more probable to cause disease than inoculation by spores (accompanied by negative mean values).

**(a)**	**Seeds + spores**	**Rhizomes + spores**	**Infected rhizomes**	**Infected rhizomes + spores**
Effect of treatment on the proportion of diseased plants: Means with 95% credible intervals				
**Ochropsora**	(-0.02) **0.00** (0.01)	(-0.04) **0.01** (0.07)	(0.16) **0.27** (0.40)	(-0.07) **0.12** (0.30)
**Tranzschelia**	(-0.01) **0.00** (0.01)	(-0.04) **0.00** (0.03)	(0.25) **0.37** (0.49)	(-0.12) **0.03** (0.18)
**Synchytrium**	(0.06) **0.10** (0.14)	(0.004) **0.06** (0.13)	(-0.04) **0.00** (0.03)	(-0.10) **-0.01** (0.11)
**Urocystis**	(-0.01) **0.00** (0.01)	(-0.01) **0.04** (0.12)	(-0.03) **0.01** (0.07)	(-0.10) **0.00** (0.12)

**(b)**	*Probability that spores cause higher disease rates than infected rhizomes*	*Means with 95% credible intervals*
**Ochropsora**	0	(-0.38) **-0.25** (-0.13)
**Tranzschelia**	0	(-0.49) **-0.37** (-0.26)
**Synchytrium**	0.98	(0.01) **0.06** (0.13)
**Urocystis**	0.83	(-0.03) **0.03** (0.11)

Table S3. Overview of our hypotheses on the spatial and temporal dynamics of Ochropsora, Tranzschelia, Synchytrium and Urocystis pathogens, as based on the life history traits of each pathogen. The last column gives the level of support we found for each hypothesis after analysing our data.

**Topic**	**Pathogen**	**Prediction**	**Hypothesis**	**Result**
**Patch occupancy**	Ochropsora, Tranzschelia	**Uncertain**	Low colonization rates and low extinction rates (see below), resulting in unpredictable patch occupancy.	**Strong support:** Synchytrium had a higher patch occupancy than Urocystis.
	Synchytrium	**High**	High colonization rates in combination with intermediate extinction rates (see below), resulting in high patch occupancy.	
	Urocystis	**Low**	Medium colonization rates in combination with high extinction rates (see below), resulting in low patch occupancy.	
**Colonization rates**	Ochropsora, Tranzschelia	**Low**	Limited colonization ability due to low probability to infect hosts via spores.	**Strong support:** On average, Synchytrium had the highest colonization rate, Urocystis intermediate colonization rate, and Ochropsora and Tranzschelia low colonization rates.
	Synchytrium	**High**	High colonization ability due to relatively high probability to infect adults and seedlings via spores.	
	Urocystis	**Medium**	Medium colonization ability due to relatively high probability to infect adult plants, but not seedlings.	
**Extinction rates**	Ochropsora, Tranzschelia	**Low**	High probability of successful overwintering due to the ability to overwinter in rhizomes.	**No support:** Extinction rates did not differ significantly among the four pathogens.
	Synchytrium	**Medium**	Not able to overwinter in the rhizomes, but can infect both adults and seedlings via spores.	
	Urocystis	**High**	Low ability to overwinter in the rhizomes, and infects adults mainly via spores.	
**Impact of host patch size on patch occupancy,**	All	**High**	For all four pathogens, we hypothesized a strong effect of host patch size on patch occupancy, colonization and extinction. Smaller host patches were hypothesized to have lower colonization rates and higher	**Moderate support**: There was a strong positive effect of host patch size on patch occupancy and colonization rates for all pathogens.

**colonization and extinction**			extinction rates, leading to lower occupancy of small host patches compared to large host patches.	However, regarding extinction rates, only Synchytrium was more likely to go extinct in smaller host patches.
**Impact of spatial connectivity on patch occupancy, colonization and extinction**	Ochropsora, Tranzschelia	**High**	Due to the low probability to infect via spores, these diseases are likely to be highly dependent on spatial connectivity in order to spread from one host patch to another via the rhizomes, affecting both colonization and rescue effects, leading to a strong positive effect of connectivity on patch occupancy.	**Weak support:** Patch occupancy by Ochropsora and Tranzschelia was, as hypothesized, positively affected by spatial connectivity in some of the years. Contrary to our hypothesis, patch occupancy by Synchytrium was negatively affected by spatial connectivity in most years. Urocystis occupancy was only affected by the interaction between spatial connectivity and host patch size. The colonization and extinction rates of the pathogens were not affected by spatial connectivity, which did not support our hypothesis.
	Synchytrium	**Low**	Due to high probability to infect via spores, and to infect seedlings as well as plants, this disease is likely less dependent on spatial connectivity in order to spread from one host patch to another, with little impact on colonization and rescue effects, leading to a weak effect of connectivity on patch occupancy.	
	Urocystis	**Low – medium**	Due to high probability to infect plants via spores, this disease is likely less dependent on spatial connectivity in order to spread from one host patch to another, with little impact on colonization and rescue effects, leading to a weak effect of connectivity on patch occupancy.	
**Intensity of disease within a host patch**	Ochropsora, Tranzschelia	**Low**	Spread of diseases via rhizomes within a host patch is slow.	**Strong support:** As hypothesized, Synchytrium had the highest disease intensities within host patches, then Urocystis, and then Ochropsora and Tranzschelia.
	Synchytrium	**High**	Disease can quickly spread via spore dispersal within a host patch, affecting plants as well as seedlings.	
	Urocystis	**Medium**	Disease can quickly spread via spore dispersal within a host patch, affecting only plants.	
**Impact of alternate host on patch occupancy**	Ochropsora	**High**	Since Ochropsora is dependent on an alternate host for the sexual part of its lifecycle, we hypothesize that occupancy by Ochropsora is highly related to presence of the alternate host.	**Strong support:** Ochropsora occupancy was higher when the alternate host was present.

Table S4. Metapopulation dynamics of Ochropsora, Tranzschelia, Synchytrium and Urocystis pathogens from 2017 to 2020, including the number of diseased host patches, patch occupancy (the proportion of diseased host patches), colonization and extinction events and rates, and the disease intensity within a host patch (mean percentage of diseased plants within host patches ± standard deviation).

	Number of diseased patches	Patch occupancy	Colonization events	Colonization rate	Extinction events	Extinction rate	Disease intensity (mean ± sd)
**2017** (n = 139 populations)							
Ochropsora	16	0.12	-	-	-	-	0.7 ± 1.0
Tranzschelia	5	0.04	-	-	-	-	0.4 ± 0.1
Synchytrium	23	0.17	-	-	-	-	8.3 ± 13.5
Urocystis	25	0.18	-	-	-	-	3.6 ± 3.0
**2018** (n = 138 populations)							
Ochropsora	16	0.12	2	0.02	2	0.13	0.4 ± 0.5
Tranzschelia	9	0.07	4	0.03	0	0	0.1 ± 0.09
Synchytrium	41	0.30	18	0.16	0	0	28.8 ± 29.0
Urocystis	24	0.17	3	0.03	4	0.16	2.3 ± 2.4
**2019** (n = 83 populations)							
Ochropsora	13	0.16	3	0.04	5	0.45	0.5 ± 0.6
Tranzschelia	8	0.10	2	0.03	2	0.25	0.3 ± 0.2
Synchytrium	31	0.37	6	0.11	4	0.14	27.3 ± 28.9
Urocystis	19	0.23	2	0.03	3	0.15	3.0 ± 2.7
**2020** (n = 81 populations)							
Ochropsora	14	0.17	2	0.03	1	0.08	0.9 ± 1.3
Tranzschelia	4	0.05	0	0	4	0.50	0.8 ± 0.3

Synchytrium	25	0.31	1	0.02	6	0.20	17.6 ± 20.5
Urocystis	21	0.26	5	0.08	2	0.11	3.6 ± 3.2
**Mean values across years**							
Ochropsora	14.8	0.14	2.3	0.03	2.7	0.22	0.6
Tranzschelia	6.5	0.07	2	0.02	2	0.25	0.4
Synchytrium	30	0.29	8.3	0.10	3.3	0.11	20.5
Urocystis	22.3	0.21	3.3	0.05	3	0.14	3.1

Table S5. Panel **(a)** shows the effect of pathogen identity on path occupancy and colonization and extinction rates. Year was included as a random effect, see table S1 for the fitted models. Panel **(b)** shows the results of pathogen-specific contrasts of the significant models, using the function *emmeans* in the R-package *emmeans*. Significant p-values (< 0.05) are presented in bold.

**(a)**	*p-value*	*df*	χ^2
Patch occupancy	**<0.001**	3	66.92
Colonization rates	**<0.001**	3	20.04
Extinction rates	0.59	3	1.93

**(b)**	**Patch occupancy**		**Colonization rates**	
	p-value	*z-value*	*p-value*	*z-value*
Ochropsora – Tranzschelia	**0.002**	3.55	0.97	0.45
Ochropsora – Synchytrium	**<0.001**	-4.81	**0.01**	-3.24
Ochropsora – Urocystis	0.12	-2.22	0.71	-1.07
Tranzschelia – Synchytrium	**<0.001**	-7.75	**0.002**	-3.60
Tranzschelia – Urocystis	**<0.001**	-5.54	0.44	-1.50
Synchytrium – Urocystis	**0.04**	2.68	0.10	2.28

Table S6. The effect of host patch size, host spatial connectivity and their interaction on patch occupancy. Host spatial connectivity accounts for the distance from the focal host patch to all other host patches, as well as the size of all host patches (text S1). For a visualization of the interactive effect between host patch size and host spatial connectivity for Urocystis, see figure S4. Significant p-values (< 0.05) are presented in bold.

	**Host patch size**		**Host spatial connectivity**		**Host patch size × host spatial connectivity**	
	p-value	χ^2	*p-value*	χ^2	*p-value*	χ^2
**2017**						
Ochropsora	**<0.001**	35.34	0.56	0.34	0.90	0.02
Tranzschelia	**<0.001**	26.95	0.59	0.29	0.82	0.05
Synchytrium	**<0.001**	29.95	**<0.001**	11.04	0.49	0.47
Urocystis	**<0.001**	19.32	0.73	0.12	**0.01**	5.99
**2018**						
Ochropsora	**<0.001**	34.36	0.57	0.32	0.89	0.02
Tranzschelia	**<0.001**	41.70	0.68	0.17	0.69	0.15
Synchytrium	**<0.001**	38.21	**0.03**	4.47	0.81	0.06
Urocystis	**<0.001**	22.07	0.33	0.96	0.05	3.80
**2019**						
Ochropsora	**<0.001**	28.76	**0.03**	4.55	0.39	0.73
Tranzschelia	**<0.001**	31.32	**0.004**	8.09	0.57	0.32
Synchytrium	**<0.001**	33.61	**0.03**	4.73	0.97	0.00
Urocystis	**0.01**	9.77	0.08	2.98	**0.02**	5.37
**2020**						
Ochropsora	**<0.001**	25.66	**0.01**	6.46	0.86	0.03
Tranzschelia [2]	**<0.001**	33.62	**<0.001**	13.56	0.78	0.08
Synchytrium	**<0.001**	17.38	0.35	0.87	0.37	0.82
Urocystis	**0.002**	10.06	0.22	1.51	0.11	2.54

[2] The KS test (*DHARMa*) detected a slight deviation in the expected distribution of the residuals of this model

Table S7. The effects of host patch size, spatial connectivity of the pathogen, and their interaction on colonization and extinction rates of Ochropsora, Tranzschelia, Synchytrium and Urocystis, from 2017 to 2020. Colonization rates were modelled as the proportion of unoccupied host patches in year *t*-1 that become occupied in year *t*. Extinction rates were modelled as the proportion of occupied host patches in year *t*-1 the became unoccupied in year *t*. Year was included as a random effect in the models. See table S1 for fitted models. Significant p-values (< 0.05) are presented in bold.

	**Host patch size**		**Pathogen spatial connectivity**		**Host patch size × pathogen spatial connectivity**	
	p-value	χ^2	*p-value*	χ^2	*p-value*	χ^2
**Colonization**						
Ochropsora	**0.004**	8.39	0.30	1.05	0.66	0.19
Tranzschelia	**<0.001**	12.15	0.19	1.71	0.95	0.00
Synchytrium	**<0.001**	11.98	0.26	1.26	0.91	0.01
Urocystis	**0.01**	6.38	0.95	0.00	0.34	0.90
**Extinction**						
Ochropsora	0.11	2.52	0.83	0.05	0.37	0.79
Tranzschelia	0.23	1.42	0.43	0.61	0.20	1.61
Synchytrium	**0.04**	4.19	0.36	0.85	0.90	0.02
Urocystis	0.96	0.00	0.62	0.25	0.62	0.24

Table S8. The effect of pathogen identity on disease intensity within a host patch. Year was included as a random effect and host patch size as a covariate, see table S1 for the fitted model. Shown are the results of pathogen-specific contrasts using the function *emmeans* in the R-package *emmeans*. Significant p-values (< 0.05) are presented in bold.

	**Disease intensity**	
	p-value	*t-value*
Ochropsora – Tranzschelia	0.90	0.69
Ochropsora – Synchytrium	**<0.001**	-14.29
Ochropsora – Urocystis	**<0.001**	-8.20
Tranzschelia – Synchytrium	**<0.001**	-11.84
Tranzschelia – Urocystis	**<0.001**	-7.19
Synchytrium – Urocystis	**<0.001**	6.44

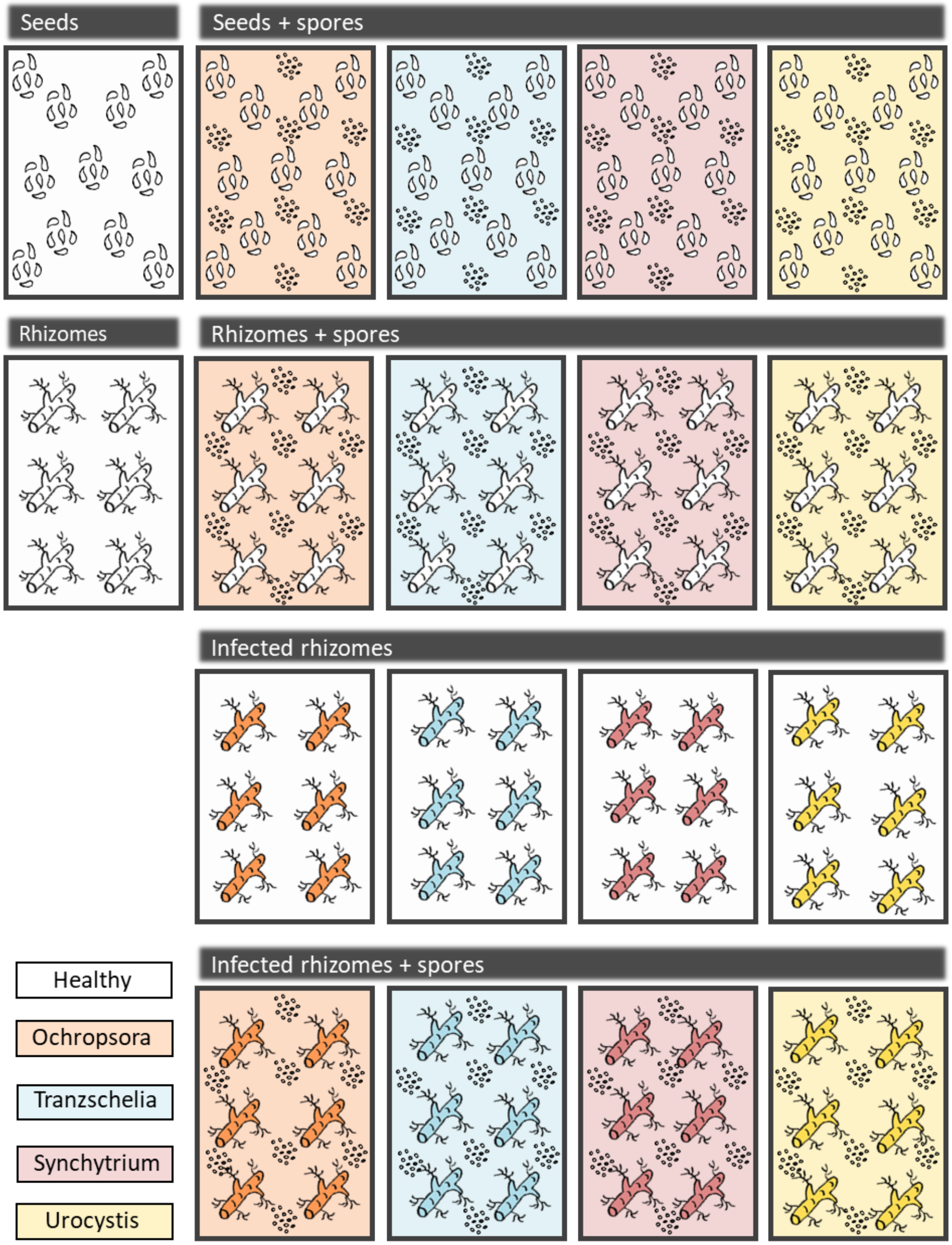

Figure S1. Pathogen treatments of seeds and rhizomes in the common garden, including “seeds”, “seeds + spores”, “rhizomes”, “rhizomes + spores”, “infected rhizomes” and “infected rhizomes + spores”. Trays coloured in white did not receive spores, trays coloured in orange, blue, red, or yellow received Ochropsora, Tranzschelia, Synchytrium or Urocystis spores, respectively. Seeds and rhizomes without previous infection are coloured white (“healthy”), and rhizomes from plants previously infected by a disease are coloured in orange (Ochropsora), blue (Tranzschelia), red (Synchytrium) or yellow (Urocystis).

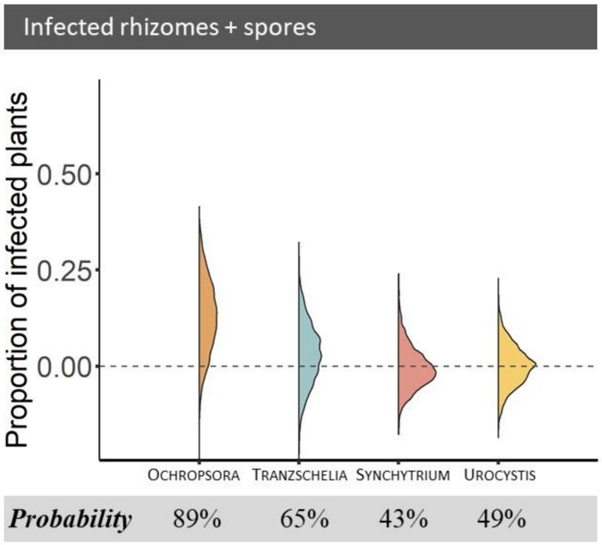

Figure S2. Half-violin plots of the posterior probability distributions, which visualize the non-additive effect of spore inoculation and previous infection on the proportion of diseased plants within trays for Ochropsora (orange), Tranzschelia (blue), Synchytrium (red) and Urocystis (yellow). Below each panel, the probability that a treatment will have more diseased plants than expected from the additive effect of spore inoculation and previous rhizome infection is given (i.e. the proportion of the probability distribution that is above zero). Probabilities above zero indicate increased susceptibility, whereas probabilities below zero indicate increased resistance. See table S1 for details of the fitted models.

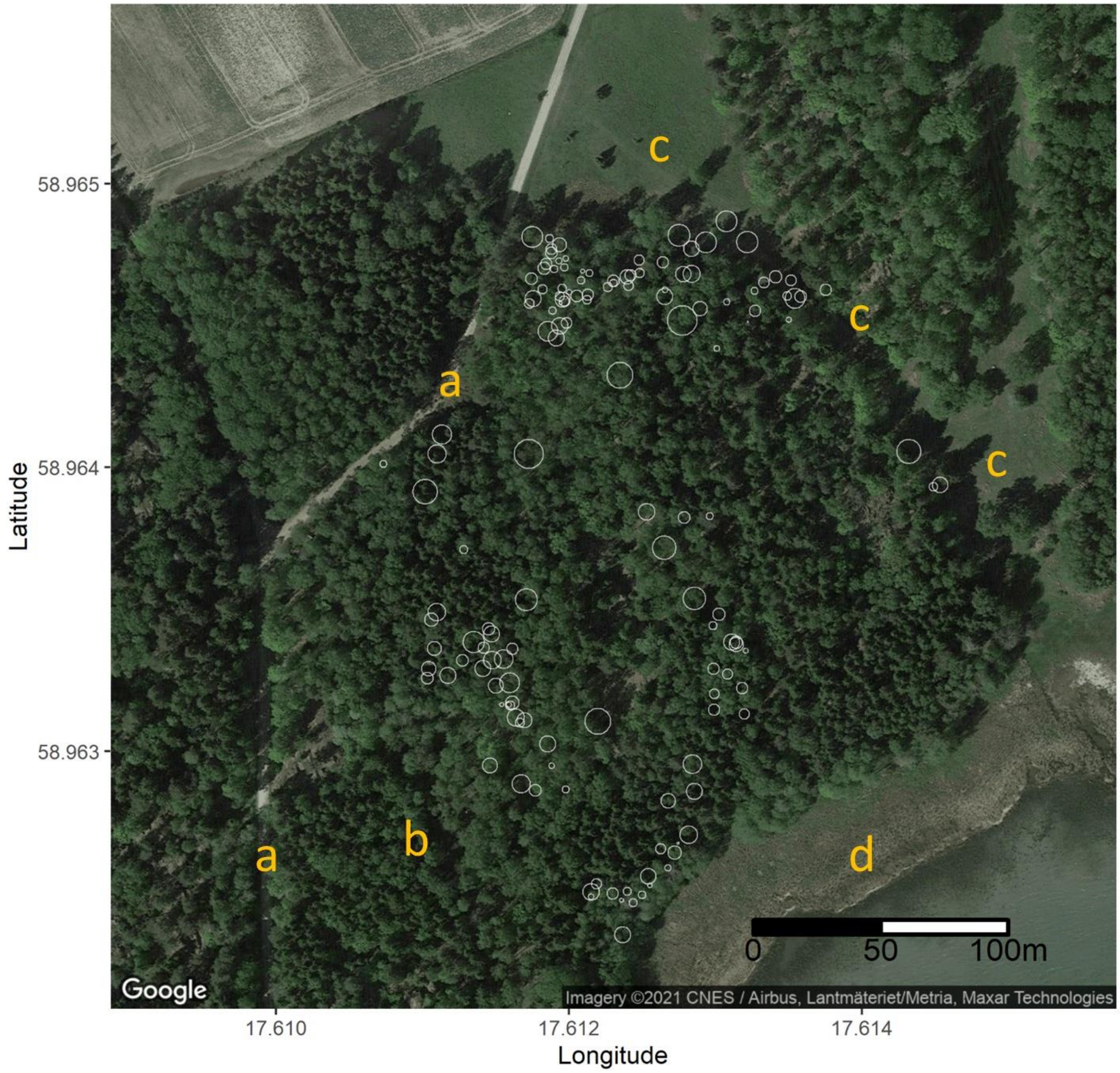

Figure S3. Map of all 139 patches of *Anemone nemorosa* in the study area within the Tullgarn nature reserve, 2017. Anemone patches are indicated as white circles, which are scaled according to the log-transformed size (length × width) of a patch. The study area was locally isolated by **(a)** a road, **(b)** a forest area without anemone patches, **(c)** a grass field without anemone patches and **(d)** the sea.

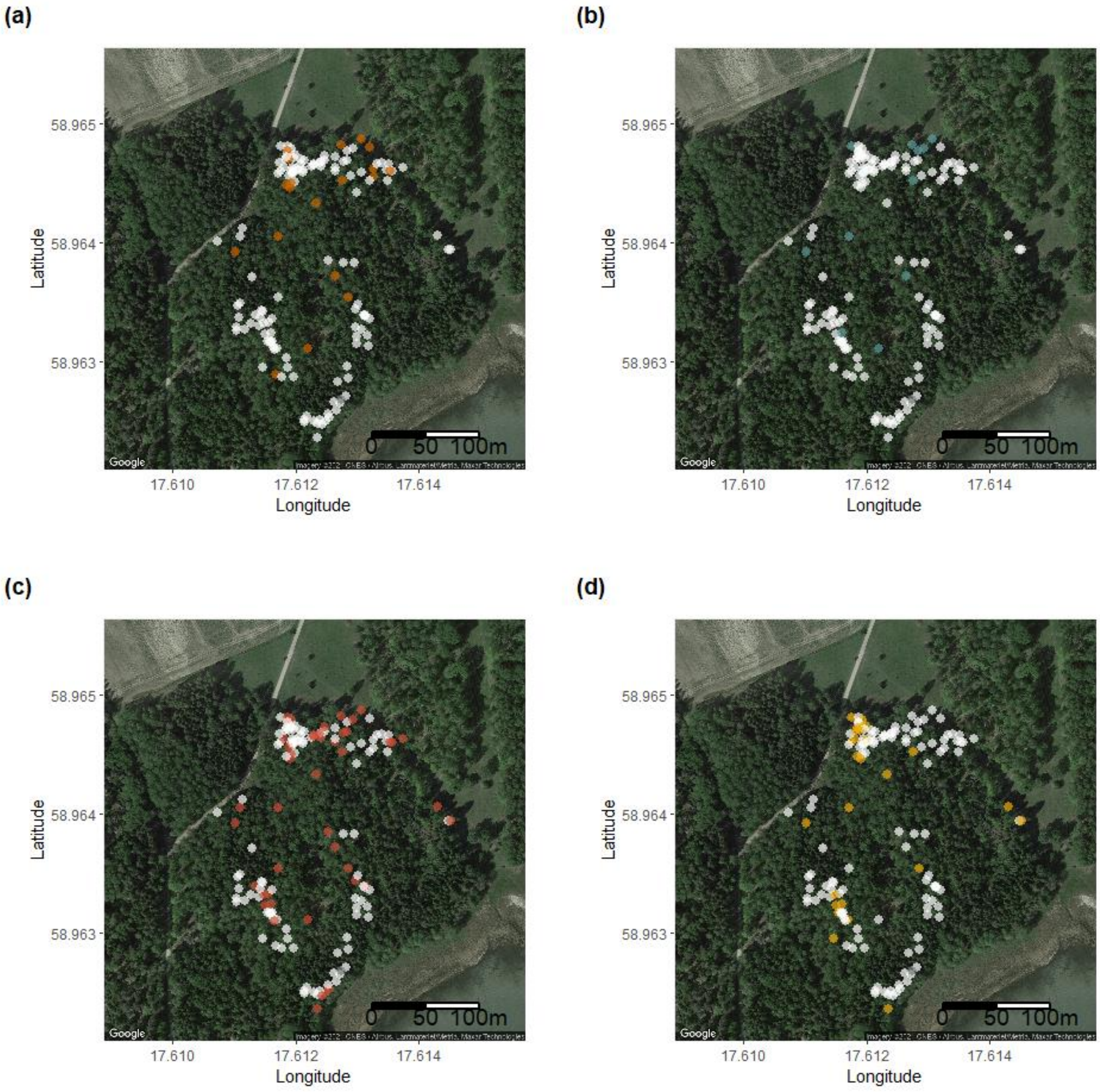

Figure S4. The disease status of all patches of *Anemone nemorosa* for **(a)** Ochropsora, **(b)** Tranzschelia, **(c)** Synchytrium and **(d)** Urocystis in the study area within the Tullgarn nature reserve, 2018. Patches without disease are indicated as white circles, and patches with disease are indicated as orange (Ochropsora), blue (Tranzschelia), red (Synchytrium) or yellow circles (Urocystis).

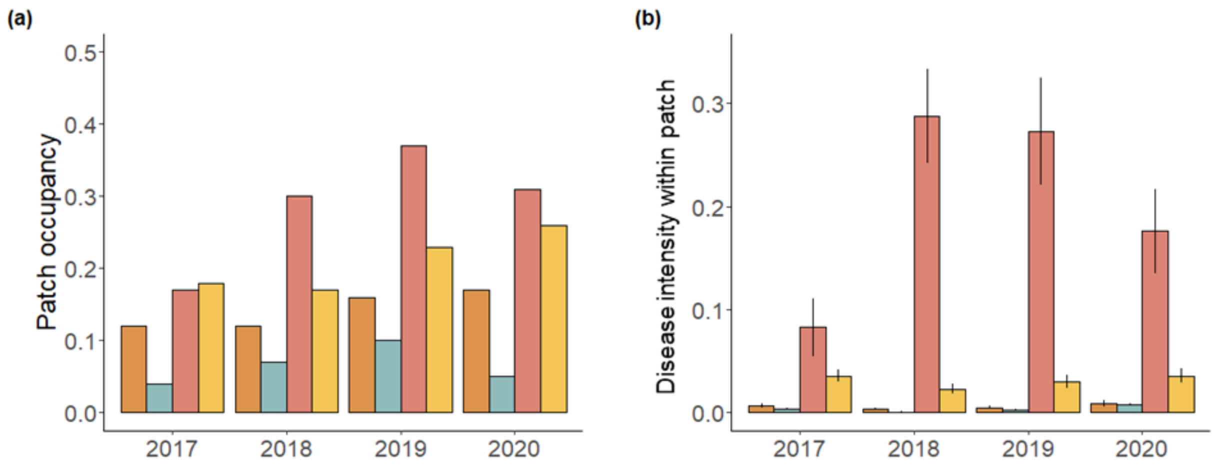

Figure S5. Patch occupancy and disease intensities within host patches in 2017, 2018, 2019 and 2020. Panel **(a)** shows patch occupancy (proportion of diseased host patches) for Ochropsora (orange), Tranzschelia (blue), Synchytrium (red) and Urocystis (yellow). Panel **(b)** shows disease intensity within a host patch, i.e. the proportion of Ochropsora-, Tranzschelia-, Synchytrium- and Urocystis-diseased plants, for all occupied host patches. Error bars present standard errors.

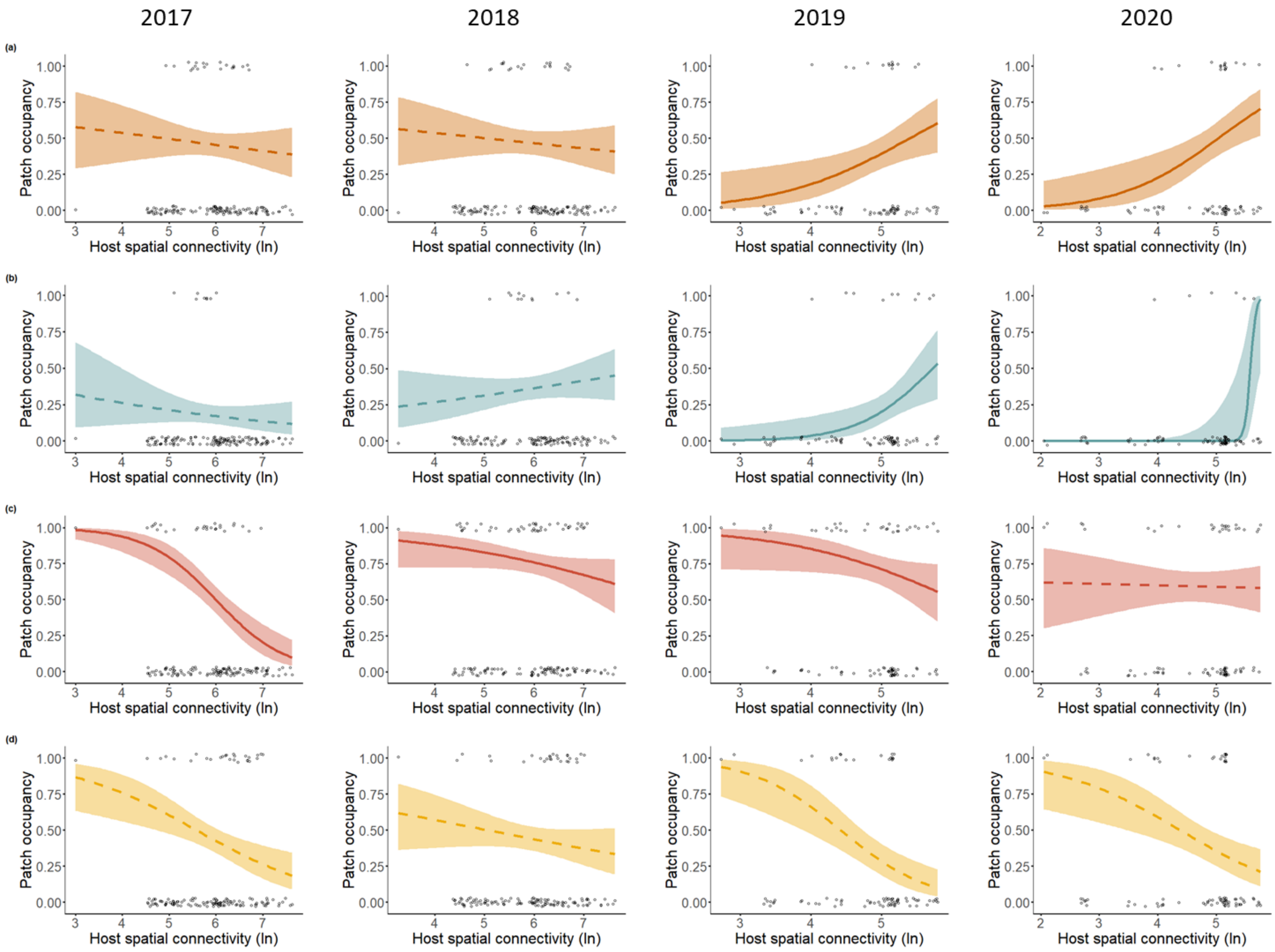

2017
2018
2019
2020
(a)
(b)
(c)
(d)
Patch occupancy
Host spatial connectivity (ln)

Figure S6. The effect of host spatial connectivity (ln-transformed) on patch occupancy for **(a)** Ochropsora, **(b)** Tranzschelia, **(c)** Synchytrium and **(d)** Urocystis in each of the survey years (2017 to 2020, from left to right). Host spatial connectivity accounts for the distance from the focal host patch to all other host patches, as well as the size of all host patches. Lines represent the predicted relationship with 95% confidence interval. Solid lines represent significant relationships ($p < 0.05$). For fitted models, see table S1, and for p-values and test statistics of the models see table S6.

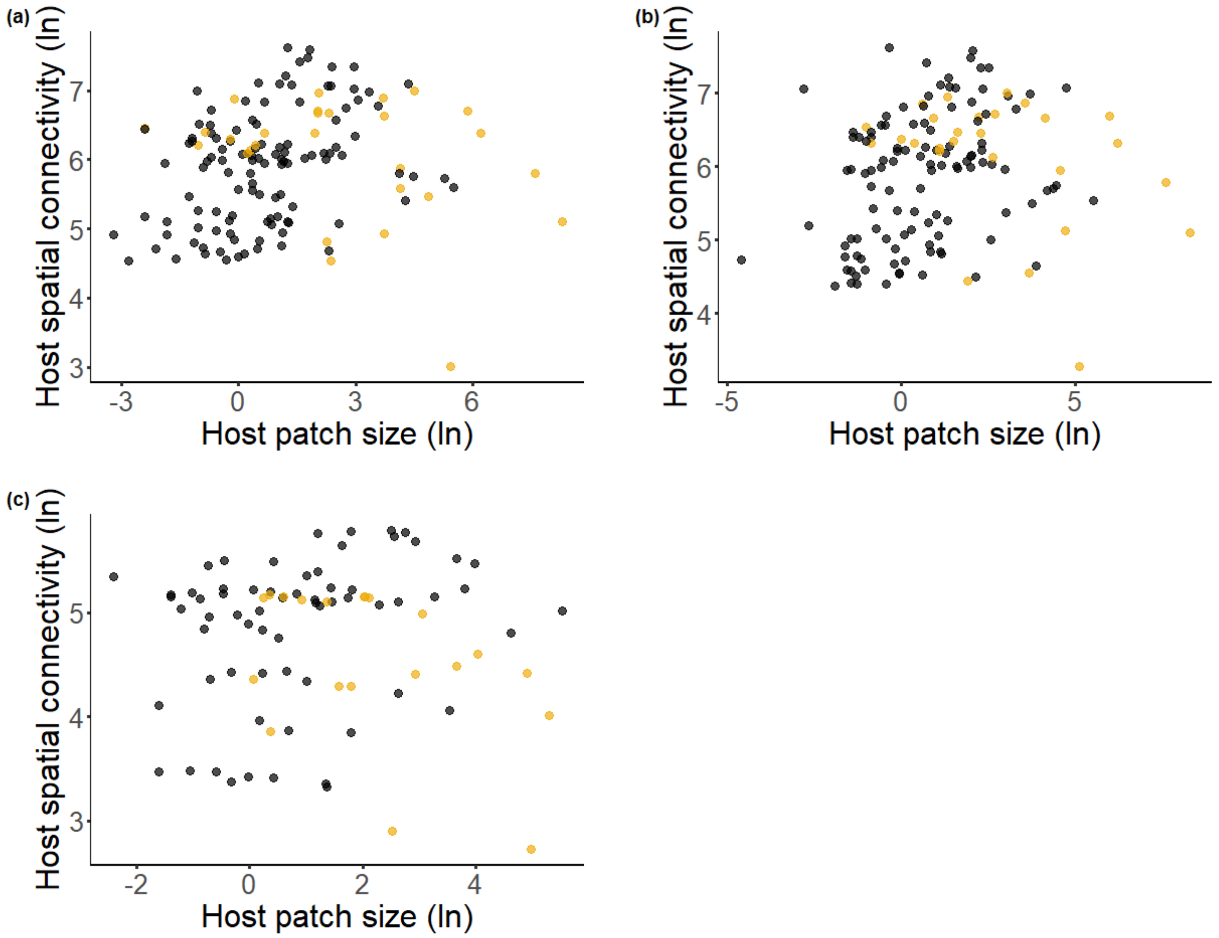

Figure S7. Interactive effect of host patch size (ln-transformed) and host spatial connectivity (ln-transformed) on incidence of Urocystis in **(a)** 2017 **(b)** 2018 and **(c)** 2019. Host spatial connectivity accounts for the distance from the focal host patch to all other host patches, as well as the size of all non-focal host patches. Yellow dots represent Urocystis-diseased patches, black dots represent patches without disease. For small host patches, Urocystis is present mostly when patches have a high connectivity, whereas larger host patches are diseased also when connectivity is low. For fitted models, see table S1, and for p-values and test statistics of the models see table S6.

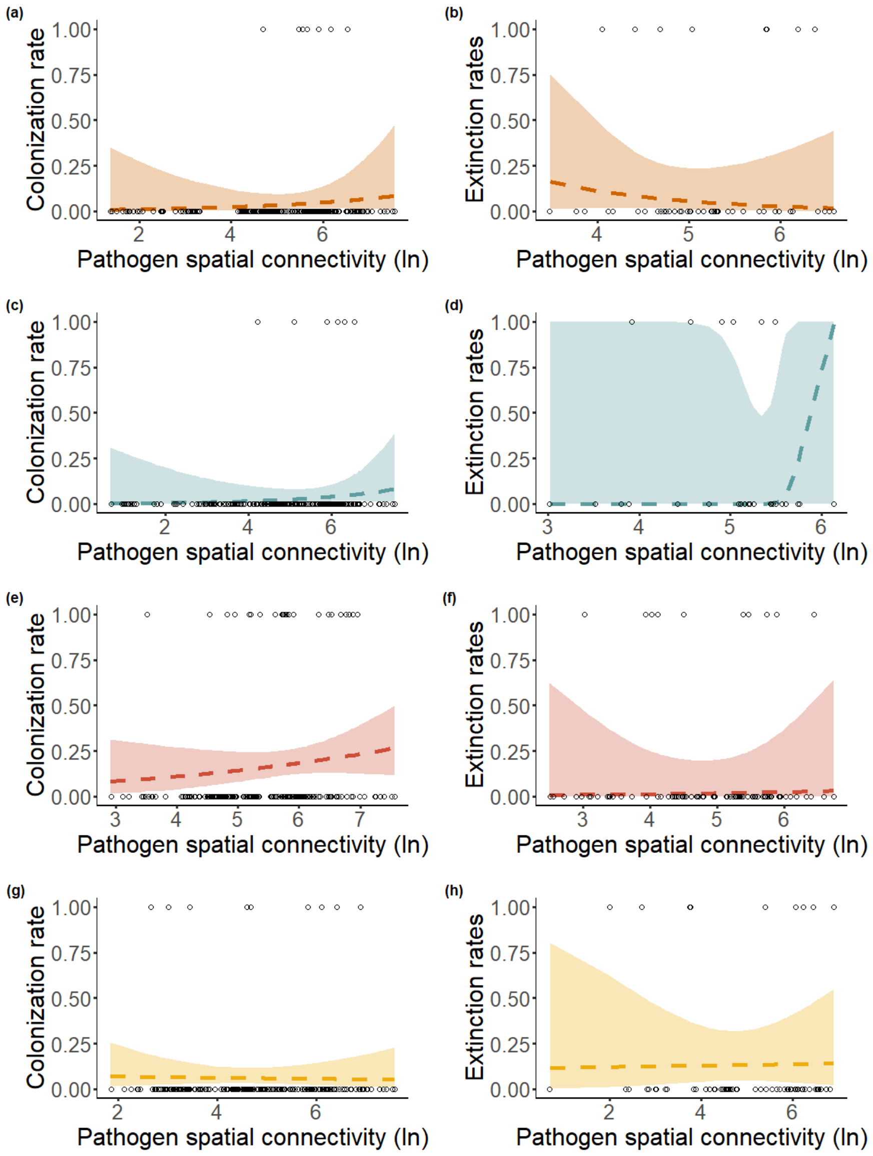

Figure S8. The effect of pathogen spatial connectivity (ln-transformed) on colonization (left) and extinction rates (right) for **(a, b)** Ochropsora, **(c, d)** Tranzschelia, **(e, f)** Synchytrium and **(g, h)** Urocystis, during all survey years (2017 – 2020). Pathogen spatial connectivity accounts for the distance from the focal host patch to all other diseased host patches, as well as the size of those host patches. For colonization rate, the y-axis represents the proportion of unoccupied host patches in year *t*-1 that become occupied in year *t*. For extinction rate, the y-axis represents the proportion of occupied host patches in year *t*-1 the became unoccupied in year *t*. Lines represent the predicted relationship with 95% confidence interval. Dashed lines represent non-significant relationships (p > 0.05). For fitted models, see table S1, and for p-values and test statistics of the models see table S7.

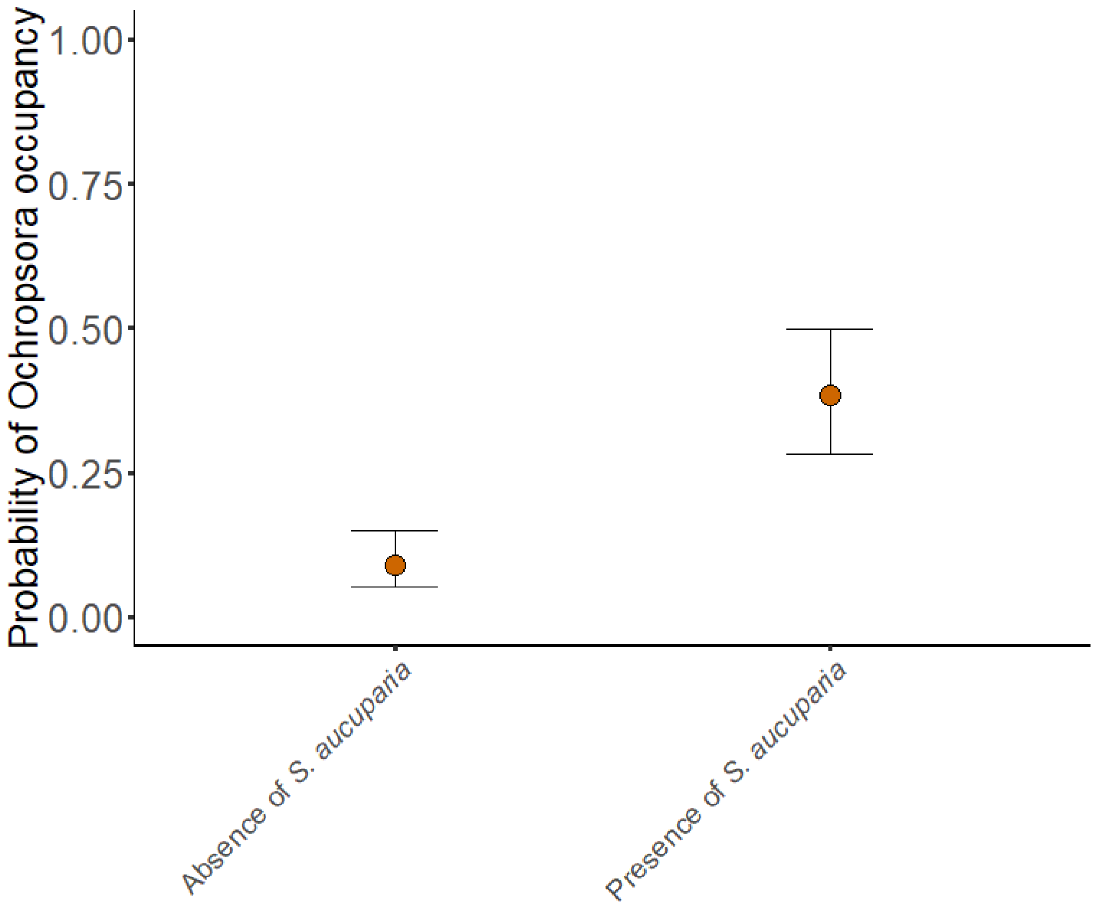

Figure S9. The relationship between presence of *Sorbus aucuparia* (within ≤ 1 meters from the host patch) and patch occupancy by Ochropsora. Shown are predicted means and confidence intervals, see table S1 for fitted model.

Methods S1. Calculation of the spatial connectivity indices.

The Spatial connectivity index of the host patches (S^H) was calculated for each year (Jousimo *et al.*, 2014), and represents the sum of all distances from the focal host patch *i* to all other host patches *j* (d_{ij}) weighed by their size (A_j, length × width of the host patch, m^2) and the inverse dispersal parameter ($\alpha = 0.02$) in year *t*:

$$S_{it}^{H} = \sum_{j \neq i} exp^{-\alpha d_{ij}} \; A_j$$

The spatial connectivity index of the pathogen (S^P) represents the sum of all distances from the focal host patch *i* to all diseased non-focal host patches *j* (p_j = disease incidence of the non-focal host patch, 0 or 1, and d_{ij} = distance from focal to non-focal host patch) weighed by their size (A_j, length × width of the host patch, m^2) and the inverse dispersal parameter ($\alpha = 0.02$) (Oksanen, 2004; Jousimo *et al.*, 2014) in year *t* - 1:

$$S_{it-1}^{P} = \sum_{j \neq i} p_j \, exp^{-\alpha d_{ij}} \; A_j$$

As we expected that colonization and extinction dynamics were more sensitive to proximity to diseased host patches (rather than any host patch irrespective of disease status), we used spatial connectivity of the pathogen when modelling colonization and extinction dynamics (Laine & Hanski, 2006; Jousimo *et al.*, 2014), while adopting the spatial connectivity of the host when modelling patch occupancy.

References

Jousimo J, Tack AJM, Ovaskainen O, Mononen T, Susi H, Tollenaere C, Laine A-L. **2014**. Ecological and evolutionary effects of fragmentation on infectious disease dynamics. *Science* **344**: 1289–1293.

Laine A-L, Hanski I. **2006**. Large-scale spatial dynamics of a specialist plant pathogen in a fragmented landscape. *Journal of Ecology* **94**: 217–226.

Oksanen J. **2004**. Incidence Function Model in R.
